# Associations of mental disorders in children with parents' subsequent mental disorders: nationwide cohort study from Finland and Denmark

**DOI:** 10.1192/bjp.2024.249

**Published:** 2026-01

**Authors:** Christian Hakulinen, Mai Gutvilig, Ripsa Niemi, Natalie C. Momen, Laura Pulkki-Råback, Petri Böckerman, Oleguer Plana-Ripoll, Kaisla Komulainen, Marko Elovainio

**Affiliations:** Department of Psychology, Faculty of Medicine, University of Helsinki, Helsinki, Finland; and Finnish Institute for Health and Welfare, Helsinki, Finland; Department of Psychology, Faculty of Medicine, University of Helsinki, Helsinki, Finland; Department of Clinical Epidemiology, Aarhus University and Aarhus University Hospital, Aarhus, Denmark; School of Business and Economics, University of Jyväskylä, Jyväskylä, Finland; Labour Institute for Economic Research LABORE, Helsinki, Finland; and IZA Institute of Labor Economics, Bonn, Germany; Department of Clinical Epidemiology, Aarhus University and Aarhus University Hospital, Aarhus, Denmark; and National Center for Register-based Research, Aarhus University, Aarhus, Denmark

**Keywords:** Register-based study, child and adolescent psychiatry, general adult psychiatry, epidemiology, data visualisation

## Abstract

**Background:**

Intergenerational transmission of mental disorders has been well established, but it is unclear whether exposure to a child's mental disorder increases parents' subsequent risk of mental disorders.

**Aims:**

We examined the association of mental disorders in children with their parents' subsequent mental disorders.

**Method:**

In this population-based cohort study, we included all individuals with children born in Finland or Denmark in 1990–2010. Information about mental disorders was acquired from national registers. The follow-up period began when the parent's eldest child was 5 years old (for ICD-10 codes F10–F60) or 1 year old (for codes F70–F98) and ended on 31 December 2019 or when the parent received a mental disorder diagnosis, died, or emigrated from Finland or Denmark. The associations of mental disorders in children with their parents' subsequent mental disorders were examined using Cox proportional hazards models.

**Results:**

The study cohort included 1 651 723 parents. In total, 248 328 women and 250 763 men had at least one child who had been diagnosed with a mental disorder. The risk of a parent receiving a mental disorder diagnosis was higher among those who had a child with a mental disorder compared with those who did not. For both parents, the hazard ratios were greatest in the first 6 months after the child's diagnosis (hazard ratio 2.04–2.54), followed by a subtle decline in the risk (after 2 years, the hazard ratio was 1.33–1.77).

**Conclusion:**

Mental disorders in children are associated with a greater risk of subsequent mental disorders among their parents. Additional support is needed for parents whose children have been recently diagnosed with a mental disorder.

It is well recognised that mental disorders cause a substantial burden for family members, especially parents.^1,2^ This mental and emotional toll may have many different sources. Mental disorders have considerable heritability,^3,4^ and both parents and their children might suffer from poor mental health owing to intergenerational mechanisms operating through genetic and environmental factors.^5,6^ On the other hand, parents may experience distress caused by exposure to their child's mental disorder; this is often accompanied by a need for additional economic resources and social support to provide care for the child. Each of these factors can adversely affect the well-being of the caregiver and other family members.^7–15^

The association between children's mental disorders and parents' mental health has been traditionally investigated from the perspective of caregivers, i.e. those taking care of a family member with a mental disorder. Most previous studies have been based on relatively small and nationally non-representative samples^10–12,15,16^ and have focused specifically on individuals caring for patients with a mental disorder. Thus, these studies do not describe the overall impact of mental disorders on both parents. Although some studies have explicitly examined the consequences of children's mental disorders for their parents, they have focused on specific disorders such as schizophrenia^17^ and autism^18^ in children; large-scale nationwide investigations across the full spectrum of mental disorders have been lacking. Using nationwide register data from Finland and Denmark, we examined whether mental disorders in children were associated with parents' subsequent risk of mental disorders. Whereas previous studies focused on only a few disorders and specifically on caregivers, we examined the population-level associations across the full spectrum of mental disorders diagnosed in childhood, adolescence and early adulthood. Interactive visualisations of the results are available at https://mentalnet.shinyapps.io/app_morbidity/.

## Method

### Study population

The study population consisted of all individuals whose children were born in Finland or Denmark between 1990 and 2010. Individuals with children who had died or emigrated from their country of birth before their first birthday were excluded. The data-sets were constructed by compiling nationwide registries (see the Supplementary material available at https://doi.org/10.1192/bjp.2024.249 for details), which were linked using unique personal identification numbers.

The Ethics Committee of the Finnish Institute for Health and Welfare approved the study plan (THL/184/6.02.01/2023§933). Data were linked with the permission of Statistics Finland (TK-53-1696-16), Statistics Denmark and the Finnish Institute of Health and Welfare. According to Finnish and Danish law, informed consent is not required for register-based studies. The present study adhered to the Strengthening the Reporting of Observational Studies in Epidemiology reporting guidelines.

### Study design

The relationship between parental risk of receiving a mental disorder diagnosis and having a child with a mental disorder diagnosis was examined. The follow-up period began when the parent's eldest child was deemed sufficiently old to be at risk of receiving a mental disorder diagnosis (5 years old for codes F10–F60 of the ICD-10; 1 year old for codes F70–F98 of the ICD-10). Follow-up ended on 31 December 2019 or when the parent received a mental disorder diagnosis, died or emigrated from Finland or Denmark. As the focus of the study was on new-onset mental disorders, parents with a history of diagnosed mental disorders between 1 January 1970 and the start of follow-up were excluded.

### Measurement of mental disorders

In Finland, mental disorders were classified using the ICD-8 from 1970 to 1986, ICD-9 from 1987 to 1995 and ICD-10 from 1996 onwards. The International Classification of Primary Care, 2nd edition (ICPC-2) was used alongside the ICD-10 in primary healthcare data, and ICPC-2 mental-health-related diagnoses were converted to corresponding ICD-10 subchapter categories.^19^ In Denmark, ICD-8 was used from 1969 to 1993 and ICD-10 from 1994 onwards.

Exposure to a child's mental disorder was defined using ICD-10 codes (and corresponding ICD-8, ICD-9 or ICPC-2 codes) and classified into nine broad categories of mental disorders based on subchapter F categories of the ICD-10 (e.g. F10–F19: ‘Mental and Behavioral Disorders Due to Psychoactive Substance Use’). For full details of diagnostic categories and the specific disorders included in each category, see Supplementary Table 1.

The primary exposure was any mental disorder of a child, defined as the first recorded ICD-10 subchapter F code (F00–F99, or corresponding ICD-8, ICD-9 or ICPC-2 codes) among children. Exposure to any mental disorder of a child was restricted to diagnoses received when the child was 1–25 years old. The date of a child's diagnosis was considered to be the date of the parent's exposure. In addition, exposure to specific mental disorders of a child was examined; this was restricted to diagnoses received when the child was 5–25 years old (F10–F60) or 1–25 years old (F70–F99). The date of a child's first diagnosis in each diagnostic category was considered to be the date of the parent's exposure to that disorder. One child could serve as the exposure for several mental disorders if they had diagnoses belonging to more than one diagnostic category. In cases where several children of the same parent received a mental disorder diagnosis within the same disorder category, the date of the earliest diagnosis among all that parent's children was considered to be the date of exposure to that disorder.

The outcome of the study was time to a parental mental disorder. The emergence of a parental mental disorder was defined as the first recorded ICD-10 subchapter F code (or its corresponding ICD-8, ICD-9 or ICPC-2 code) after the start of follow-up.

### Covariates

Parents' education was measured at the beginning of follow-up and further aggregated into the following three categories: primary education, secondary education and higher education. Parents' age at the birth of their first child was categorised into the following seven categories: below 20, 20–24, 25–29, 30–34, 35–39, 40–44 and above 45 years.

### Statistical analyses

Hazard ratios for a parent's mental disorder diagnosis in relation to the parent having a child with a mental disorder were examined using Cox proportional hazards models, with time since the beginning of follow-up as the underlying timescale. The follow-up period began when the parent's eldest child was either 5 years old (when ICD-10 diagnoses F10–F60 were used as the exposure) or 1 year old (when ICD-10 diagnoses F70–F98 were used as the exposure). Exposure to a child's mental disorder was treated as time-varying, such that each parent was considered to have been unexposed until their child received a mental disorder diagnosis. Moreover, to account for the hazard ratio of a parent's mental disorder depending on time since the child's mental disorder, time-dependent hazard ratios were modelled with five different time periods after the child's diagnosis examined (>0–6 months, >6–12 months, >1–1.5 years, >1.5–2 years or >2 years). When hazards were not proportional, we interpreted the hazard ratios as average ratios over the time period.^20^ Separate models were estimated for men and women and for different categories of a child's mental disorder. Finnish and Danish data were analysed separately using the same analytical strategy.

All models were adjusted for the year the follow-up began to account for calendar time. In addition, models were adjusted for the parent's age at the birth of their first child and the parent's educational attainment at the beginning of follow-up. When examining exposure to specific mental disorder categories, we also conducted analyses with additional adjustment for prior exposure to each mental disorder category except the exposure at hand. For example, when exposure to a child's mood disorder was examined, it was possible that a child of the same parent could have received an anxiety diagnosis before the date of the mood disorder. This was adjusted for in the comorbidity-adjusted models. For further details on comorbidity adjustment, see the Supplementary material.

As a sensitivity analysis, we gauged patterns in parents' HRs before the child's diagnosis to examine whether temporal patterns could be confounded by natural changes due to ageing or shared genetic and environmental factors rather than reflecting associations with the onset of the child's mental disorder. In addition to the aforementioned five time periods after the child's diagnosis, two new time periods were examined: from 12 to 6 months before the child's diagnosis, and the 6 months preceding the child's diagnosis. Importantly, the study population in the sensitivity analyses differed slightly from that used for the main models, as the beginning of follow-up was moved back by a year, resulting in differences in the individuals excluded from the study population. Last, we replicated the main analyses using only Finnish secondary care data.

All data were analysed using Stata v. 17.0 (StataCorp) and R v. 4.2.2 (R Core Team, 2022) with packages gtsummary (v. 1.6.2), tidyverse (v.1.3.2) and shiny (v.1.7.5).^21–23^

## Results

The numbers of men and women at risk of receiving a new-onset mental disorder at the start of follow-up and those excluded owing to a mental disorder diagnosis preceding the start of follow-up are reported in Table 1. Overall, 248 328 women and 250 763 men had at least one child who was diagnosed with a mental disorder. The baseline characteristics of the study population in Finland and Denmark are shown in Table 2.

**Table 1:** Frequencies of individuals excluded owing to a history of mental disorder diagnosis, being at risk at the start of follow-up, having been diagnosed or otherwise censored during follow-up, or having been exposed to at least one child diagnosed with a mental disorder during follow-up

Details of study population	Finland		Denmark	
	Women	Men	Women	Men
	*N* (%)	*N* (%)	*N* (%)	*N* (%)
Excluded owing to a history of mental disorder diagnosis				
Follow-up at 1^a^	20 586 (5%)	23 237 (5.8%)	20 577 (4.5%)	13 462 (3%)
Follow-up at 5^a^	32 119 (7.8%)	31 868 (8%)	30 317 (6.7%)	20 570 (4.7%)
At risk at start of follow-up				
Follow-up at 1^b^	391 902 (100%)	379 854 (100%)	441 195 (100%)	438 772 (100%)
Follow-up at 5^b^	377 452 (100%)	368 094 (100%)	420 208 (100%)	419 488 (100%)
Mental disorder during follow-up				
Follow-up at 1^b^	104 510 (26.7%)	67 318 (17.7%)	51 313 (11.6%)	39 384 (9%)
Follow-up at 5^b^	92 977 (24.6%)	58 687 (15.9%)	41 573 (9.9%)	32 276 (7.7%)
Emigrated during follow-up				
Follow-up at 1^b^	7766 (2%)	7583 (2%)	24 212 (5.5%)	29 087 (6.6%)
Follow-up at 5^b^	5170 (1.4%)	5502 (1.5%)	13 405 (3.2%)	18 005 (4.3%)
Died during follow-up				
Follow-up at 1^b^	3119 (0.8%)	7890 (2.1%)	6137 (1.4%)	11 592 (2.6%)
Follow-up at 5^b^	2798 (0.7%)	6842 (1.9%)	5697 (1.4%)	10 498 (2.5%)
At least one child diagnosed with a mental disorder during follow-up				
Any mental disorder (follow-up at 1)^b^	155 844 (39.8%)	157 466 (41.5%)	92 484 (21%)	93 297 (21.3%)
Substance use disorders (follow-up at 5)^b^	16 804 (4.5%)	16 991 (4.6%)	6600 (1.6%)	6433 (1.5%)
Psychotic disorders (follow-up at 5)^b^	4770 (1.3%)	4953 (1.3%)	8540 (2%)	8439 (2%)
Mood disorders (follow-up at 5)^b^	43 635 (11.6%)	45 673 (12.4%)	19 990 (4.8%)	20 132 (4.8%)
Anxiety disorders (follow-up at 5)^b^	63 711 (16.9%)	66 467 (18.1%)	42 526 (10.1%)	43 109 (10.3%)
Eating disorders (follow-up at 5)^b^	8564 (2.3%)	8821 (2.4%)	8565 (2%)	8527 (2%)
Personality disorders (follow-up at 5)^b^	3046 (0.8%)	3283 (0.9%)	8077 (1.9%)	8131 (1.9%)
Intellectual disabilities (follow-up at 1)^b^	6190 (1.6%)	6315 (1.7%)	6489 (1.5%)	6515 (1.5%)
Developmental disorders (follow-up at 1)^b^	8005 (2%)	8436 (2.2%)	21 479 (4.9%)	21 925 (5%)
Childhood-onset disorders (follow-up at 1)^b^	65 301 (16.7%)	68 043 (17.9%)	38 821 (8.8%)	39 619 (9%)

a. Percentage of the population before exclusion.

b. Percentage of individuals at risk when follow-up started.

Follow-up at 1, the follow-up period began when the eldest child was 1 year old; follow-up at 5, the follow-up period began when the eldest child was 5 years old.

**Table 2:** Characteristics of study population

Characteristics	Finland				Denmark			
	Women		Men		Women		Men	
	Follow-up started at 1	Follow-up started at 5	Follow-up started at 1	Follow-up started at 5	Follow-up started at 1	Follow-up started at 5	Follow-up started at 1	Follow-up started at 5
	Median (IQR)	Median (IQR)	Median (IQR)	Median (IQR)	Median (IQR)	Median (IQR)	Median (IQR)	Median (IQR)
Parent's birth year, *N* (%)								
<1950	731 (0.2%)	721 (0.2%)	4066 (1.1%)	3940 (1.1%)	385 (<0.1%)	364 (<0.1%)	4236 (1.0%)	3984 (0.9%)
1951–1960	29 525 (7.5%)	28 931 (7.7%)	50 765 (13%)	49 636 (13%)	26 208 (5.9%)	25 122 (6.0%)	54 404 (12%)	52 234 (12%)
1961–1970	187 879 (48%)	183 394 (49%)	194 441 (51%)	190 066 (52%)	214 203 (49%)	205 928 (49%)	231 417 (53%)	222 466 (53%)
1971–1980	151 235 (39%)	144 430 (38%)	115 721 (30%)	111 118 (30%)	182 556 (41%)	173 107 (41%)	139 032 (32%)	132 193 (32%)
>1980	22 532 (5.7%)	19 976 (5.3%)	14 861 (3.9%)	13 334 (3.6%)	17 843 (4.0%)	15 687 (3.7%)	9683 (2.2%)	8611 (2.1%)
Parent's age at start of follow-up, years	29 (25, 32)	33 (29, 36)	30 (27, 34)	34 (31, 38)	28 (26, 31)	32 (30, 35)	30 (27, 34)	34 (31, 38)
Parent's age at end of follow-up, years	48 (42, 53)	48 (42, 53)	50 (44, 55)	50 (45, 55)	50 (44, 55)	50 (45, 55)	52 (46, 57)	52 (47, 57)
Parent's education at start of follow-up, *N* (%)								
Lower secondary or less	55 753 (14%)	43 879 (12%)	69 064 (18%)	59 403 (16%)	94 763 (22%)	79 931 (19%)	93 656 (22%)	83 688 (20%)
Upper secondary	154 370 (39%)	144 042 (38%)	181 467 (48%)	171 507 (47%)	224 569 (52%)	212 286 (51%)	250 044 (58%)	240 138 (58%)
Post-secondary or tertiary	181 779 (46%)	189 531 (50%)	129 323 (34%)	137 184 (37%)	115 973 (27%)	125 501 (30%)	88 209 (20%)	92 133 (22%)
Child's age at substance use disorder diagnosis, years		18 (16, 21)		18 (16, 21)		19 (17, 22)		19 (17, 22)
Child's age at psychotic disorder diagnosis, years		18 (16, 21)		18 (16, 21)		18 (16, 21)		18 (16, 21)
Child's age at mood disorder diagnosis, years		17 (15, 20)		17 (15, 20)		18 (16, 21)		18 (16, 21)
Child's age at anxiety disorder diagnosis, years		17 (14, 20)		17 (14, 20)		16 (14, 19)		16 (14, 19)
Child's age at eating disorder diagnosis, years		16 (14, 18)		16 (14, 18)		16 (14, 19)		16 (14, 19)
Child's age at personality disorder diagnosis, years		20 (19, 22)		20 (19, 22)		19 (17, 22)		19 (17, 22)
Child's age at intellectual disability diagnosis, years	7 (5, 13)		7 (5, 13)		11 (6, 16)		12 (7, 16)	
Child's age at developmental disorder diagnosis, years	9 (6, 13)		9 (6, 13)		12 (8, 15)		12 (8, 15)	
Child's age at childhood-onset disorder diagnosis, years	9 (6, 13)		9 (6, 13)		12 (8, 15)		12 (8, 15)	
Child's age at first mental disorder diagnosis, years	12 (6, 17)		12 (6, 17)		15 (10, 18)		15 (10, 18)	

Follow-up at 1, the follow-up period began when the eldest child was 1 year old; follow-up at 5, the follow-up period began when the eldest child was 5 years old; IQR, interquartile range.

**Fig. 1:**
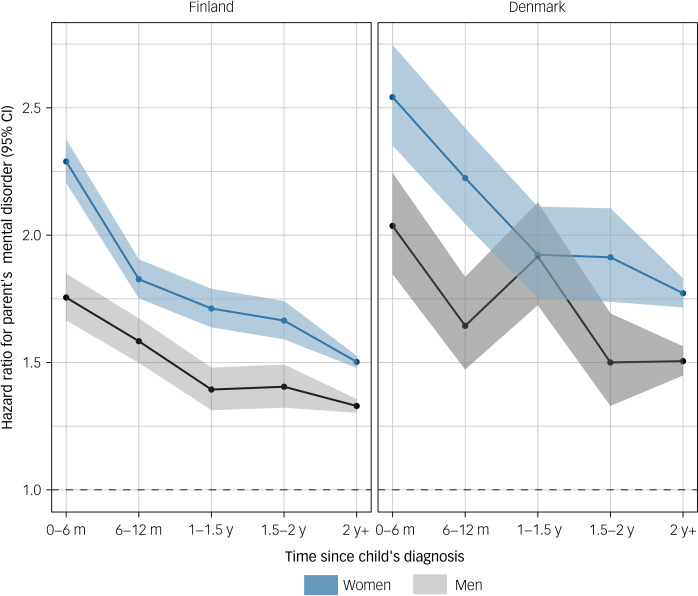
Hazard ratios and 95% confidence intervals for parents' mental disorder in relation to any mental disorder of a child according to time since the child's diagnosis.

The time-dependent hazard ratios for a parent receiving a mental disorder diagnosis depending on time since a child received a diagnosis of any mental disorder are shown in Fig. 1. The rate of mental disorder diagnosis was higher in parents who had a child with a mental disorder compared with those who did not. Overall, the HR was higher for women than men. Compared with parents without a child with a mental disorder, HRs for parents who had a child with a mental disorder were at their highest in the first 6 months following the child's diagnosis over the follow-up period. This was observed for both sexes (Finland, women: hazard ratio, 2.29 [95% CI, 2.21–2.38], men: hazard ratio, 1.75 [95% CI, 1.67–1.85]; Denmark, women: hazard ratio, 2.54 [95% CI, 2.35–2.75], men: hazard ratio, 2.04 [95% CI, 1.85–2.24]). The hazard ratios then gradually declined among women in both Finland and Denmark, and among men in Finland. In Denmark, hazard ratios among men decreased during the 6–12 months following the child's diagnosis, then increased between 1 and 1.5 years (hazard ratio, 1.92 [95% CI, 1.72–2.13]) before decreasing again. Regardless of temporal trends, HRs remained positive and significant after 2 years since the child's diagnosis across both sexes in Finland and Denmark.

**Fig. 2:**
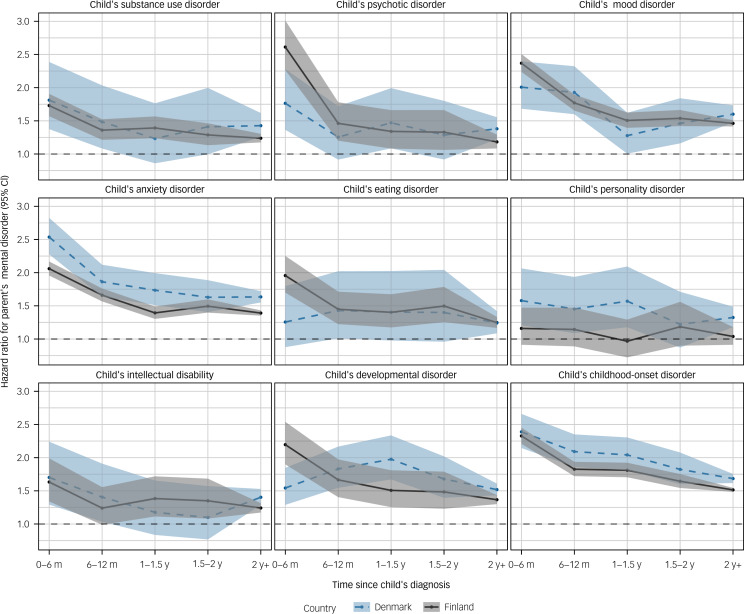
Hazard ratios and 95% confidence intervals for women's mental disorder in relation to a child's specific mental disorder according to time since the child's diagnosis.

Figure 2 shows the comorbidity-adjusted hazard ratios of women receiving any mental disorder diagnosis in relation to their child receiving a specific mental disorder diagnosis. Notable exceptions to the general trend – where hazard ratios were most elevated in the year following the child's diagnosis, followed by a subtle decline during follow-up – were personality disorders in both Finland and Denmark, and eating and developmental disorders in Denmark; for these, no time-dependent associations between child's and parent's mental disorders were observed. Overall, the time-dependent associations were more pronounced among women in Finland than those in Denmark. Compared with those of the minimally adjusted models, the point estimates of the comorbidity-adjusted models were generally lower (Supplementary Table 2).

**Fig. 3:**
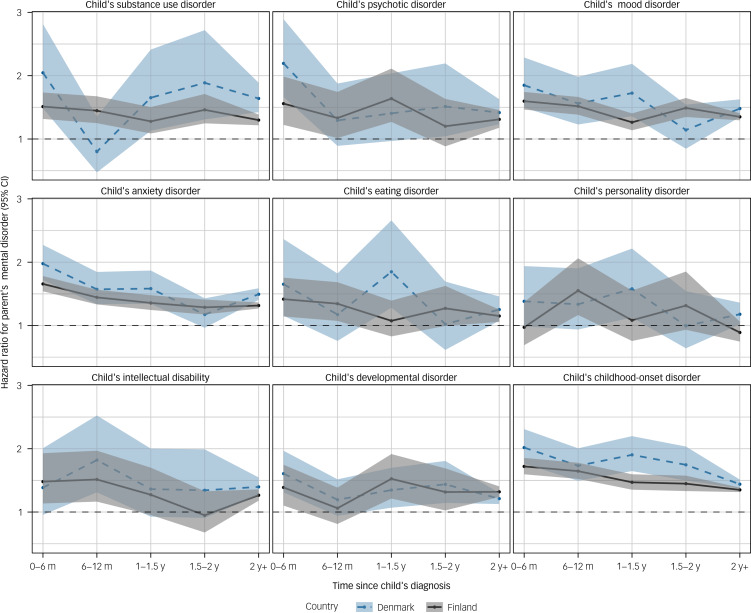
Hazard ratios and 95% confidence intervals of men's mental disorder in relation to a child's specific mental disorder according to time since the child's diagnosis.

For men, the hazard ratios for receiving a mental disorder diagnosis in relation to a child having a specific mental disorder are shown in Fig. 3. A time-dependent association, in which hazard ratios were at their highest in the 6 months following a child's diagnosis and then declined, was consistently observed for parents of children diagnosed with anxiety disorders in both Finland and Denmark. Otherwise, the temporal patterns were inconsistent and differed between Finland and Denmark, although point estimates and confidence intervals largely overlapped. The estimates from the comorbidity-adjusted models were generally lower than those from the minimally adjusted models (Supplementary Table 3).

Details of the size and characteristics of the study population used in the sensitivity analyses are reported in Supplementary Tables 4 and 5. In the sensitivity analyses, the hazard ratio of a parent receiving a mental disorder diagnosis was already higher in the 12 to 6 months before the child's diagnosis date (Supplementary Fig. 1). The hazard ratios increased sharply in the 6 months before the child's diagnosis, reaching levels comparable with those observed in the 6 months post-diagnosis. The comorbidity-adjusted hazard ratios for receiving a mental disorder diagnosis in relation to a child's specific mental disorder are shown in Supplementary Fig. 2 and Supplementary Table 6 for women and in Supplementary Fig. 3 and Supplementary Table 7 for men. Among women, increased hazard ratios in the year preceding the child's diagnosis were observed across most diagnostic categories. Among men, the temporal patterns were largely inconsistent and varied between Finland and Denmark. Finally, in the sensitivity analyses using only Finnish secondary healthcare data, the hazard ratios for a parent receiving a mental disorder diagnosis were higher than those when the primary care data were also included (Supplementary Fig. 4). Differences were observed especially among women (Supplementary Fig. 4), and specifically for mood, anxiety, developmental and childhood-onset disorders (Supplementary Fig. 5). For men, the diagnosis-specific results were more mixed (Supplementary Fig. 6).

## Discussion

In this study of two nationwide cohorts including more than 1.6 million parents from Finland and Denmark, we found that the risk of a parent receiving a mental disorder diagnosis was higher among those who had a child with a mental disorder compared with those who did not. Overall, among both women and men, the risk was at its highest in temporal proximity to the child's mental disorder diagnosis and then declined over time. When specific disorders of a child were examined, a similar time-dependent trend was generally observed across most diagnostic categories in women, whereas the temporal patterns in men were less consistent.

To our knowledge, the present study is the most comprehensive investigation of how children's mental disorders are associated with those of their parents. Although several studies have linked children's mental disorders with parents' mental health and overall well-being,^7–12^ most of these previous studies were based on small samples^10–12,15,16^ or focused on specific disorders.^17,18^ Here, utilisation of nationwide registers provided comprehensive information for two entire populations, allowing us to examine the relationship between mental disorders in children and their parents with a greater precision than previously.

Having a child diagnosed with any mental disorder was consistently associated with a greater risk of a parent receiving a subsequent mental disorder diagnosis throughout the follow-up. To a large extent, this is likely to be explained by genetic and environmental factors shared between children and their parents. However, we excluded parents with pre-existing mental disorders, and the markedly higher relative risk observed in temporal proximity to the child's diagnosis, followed by a decline, suggests that the onset of a child's mental disorder is associated with a transient increase in parents' risk of a mental disorder. The increase could already be observed 6 months before the child's diagnosis, which was not unexpected given the diagnostic delay associated with mental disorders^24–26^. Most children are likely to have experienced symptom onset before the diagnosis; thus, the demands for parental adaptation are expected to precede diagnostic confirmation of a child's disorder. The relative risk remained at its highest during the 6 months following the child's diagnosis and then started to decline. This could feasibly have resulted from the immediate psychological strain and reallocation of family resources associated with the onset of a child's mental disorder or providing care and support for the child, followed by a phase of psychological adaptation.^27^

In contrast to the analyses including any diagnosis of a child, results from analyses limited to specific disorders of a child differed across parents' sex. Whereas the temporal patterns were largely mixed among men, a time-dependent trend of markedly higher relative risk close to the time of the child's diagnosis was observed among women across most of the children's diagnostic categories. This temporal pattern was generally robust after adjustment for other mental disorders of siblings or of the children themselves, although the estimates were attenuated by these adjustments. It is important to note that in the analyses including any diagnosis of a child, only the first diagnosis among children of a parent was defined as the exposure, whereas in the disorder-specific analyses, other diagnoses could have occurred already. As such, our findings suggest that the transient elevation in the relative risk may occur in women regardless of prior comorbidity among their children, whereas for men, it is consistently observable only in relation to the first diagnosis in their family. In addition, the relative risks associated with a child's mental disorder were generally greater for women than for men. We propose that these sex differences may stem from societal and cultural expectations assigning primary caregiving roles to women,^28^ or from gendered norms that regulate experience and expression of distress.^29^

Although the results obtained with the Finnish and the Danish data were generally consistent, we also observed differences. For instance, the transient increase in the relative risk associated with a child's anxiety disorder was markedly greater in Denmark than in Finland. By contrast, there was a clear time-dependent trend associated with child eating disorders among women in Finland, but this was not observed in Denmark. Although the Finnish and Danish healthcare systems are similar in many respects,^30^ we cannot rule out potential differences in diagnostic or treatment protocols, or cultural dissimilarity affecting perceptions of mental disorders. In addition, the coverage of the Finnish and Danish data was different; whereas the Finnish data included diagnoses from both primary and secondary healthcare, the Danish data were limited to secondary healthcare only. Given that our sensitivity analyses using only Finnish secondary healthcare data suggested that the relative risk of a parent receiving a mental disorder diagnosis was higher than when the primary care data were also included, the differences in coverage between Finnish and Danish data can be considered to affect our findings.

### Limitations

The present study had limitations. First, in our study population, parents could be considered to have been exposed only if their child had received a mental disorder diagnosis in the primary or secondary healthcare system. Although individuals with severe mental disorders are likely to eventually present to the healthcare system, those whose mental disorders were untreated (or were treated only within primary healthcare in Denmark) during the follow-up were misclassified as not having a mental disorder. Studies have shown that register-based diagnoses have good validity,^31–34^ but not all disorders have been validated, and the validity of diagnosis may differ in different clinical settings. Second, as discussed above, a diagnosis is not an unambiguous ‘time zero’ for the onset of a child's mental disorder. As no information on symptoms before the diagnosis is systematically available through registers, we were not able to estimate temporal trends in parents' morbidity since the onset of the child's symptoms. Similarly, we had no information on children's remission or recovery, which could modify the associations with parents' morbidity over time. Third, we did not apply any specific residency criteria to ensure that parents were living in Finland or Denmark during the period when prior mental disorders were assessed. This may have led to shorter exclusion periods among migrant parents, potentially resulting in the parent's diagnosis during follow-up being the first one in Finland or Denmark rather than their first mental disorder diagnosis. Fourth, this was an observational study, and it is difficult to establish causality from its findings. Although adjustment for prior psychiatric comorbidity among children within a family and examination of the time before the child's diagnosis may capture some confounding shared among family members, we cannot rule out the possibility of unmeasured or residual confounding. Owing to shared unmeasured risk factors, the true associations may have been be smaller than those observed. On the other hand, residual confounding, i.e. that due to measurement error in confounders, could bias the associations in either direction. Finally, the study populations were from two Nordic countries with universal access to healthcare and strong welfare systems, which limits the generalisability of our findings. In conclusion, nationwide data from Finland and Denmark suggest that children's mental disorders may temporarily increase their parents' risk of mental disorders. These findings need to be further evaluated in different populations.

## Supporting information

Hakulinen et al. supplementary material Hakulinen et al. supplementary material

## Data Availability

Data for the present study are the property of Statistics Finland, the Finnish Institute of Health and Welfare, Statistics Denmark and the Danish Health Data Authority. The data are available from these authorities, but restrictions apply.
