# Supplementary material for: The Associations of Mental Disorders in Children With Parents’ Subsequent Mental Disorders: A Nationwide Cohort Study From Finland and Denmark
Source: Br J Psychiatry. Author manuscript; Available in PMC 2026 Jan 1. (PMC7617398; doi:10.1192/bjp.2024.249)
Supplement: Supplementary file 1 [file EMS200078-supplement-Supplementary_Materials.pdf]

ONLINE SUPPLEMENT

The Associations of Mental Disorders in Children With Parents' Subsequent Mental Disorders: A Nationwide Cohort Study From Finland and Denmark

Appendix

Description of Study Cohorts......2

Description of the Comorbidity Adjustment......3

Supplement Table 1. Diagnostic Categories of Mental Disorders in Finland and Denmark...4

Supplement Table 2. Women's Minimally and Comorbidity Adjusted Hazard Ratios and 95% Confidence Intervals for Mental Disorders in Relation to a Child's Specific Mental Disorder According to Time Since the Child's Diagnosis......6

Supplement Table 3. Men's Minimally and Comorbidity Adjusted Hazard Ratios and 95% Confidence Intervals for Mental Disorders in Relation to a Child's Specific Mental Disorder According to Time Since the Child's Diagnosis......8

Supplement Table 4. Frequencies of Persons Diagnosed Prior to Follow-up, at Risk at Start of Follow-up, Diagnosed or Otherwise Censored During Follow-up, and Exposed During Follow-up in the Sensitivity Analyses. 10

Supplement Table 5. Descriptives of Study Population in Sensitivity Analyses......11

Supplement Table 6. Women's Minimally and Comorbidity Adjusted Hazard Ratios and 95% Confidence Intervals for Mental Disorders in Relation to a Child's Specific Mental Disorder Before and After the Child's Diagnosis......12

Supplement Table 7. Men's Minimally and Comorbidity Adjusted Hazard Ratios and 95% Confidence Intervals for Mental Disorders in Relation to a Child's Specific Mental Disorder Before and After the Child's Diagnosis......14

Supplement Figure 1. Hazard Ratios and 95% Confidence Intervals of Parents' Mental Disorder in Relation to Any Mental Disorder of a Child Before and After the Child's Diagnosis......16

Supplement Figure 2. Women's Hazard Ratios and 95% Confidence Intervals of Mental Disorder in Relation to a Child's Specific Mental Disorder Before and After the Child's Diagnosis......17

Supplement Figure 3. Men's Hazard Ratios and 95% Confidence Intervals of Mental Disorder in Relation to a Child's Specific Mental Disorder Before and After the Child's Diagnosis......18

Supplement Figure 4. Hazard Ratios and 95% Confidence Intervals of Parents' Mental Disorder in Relation to Any Mental Disorder After the Child's Diagnosis in Finland......19

Supplement Figure 5. Women's Hazard Ratios and 95% Confidence Intervals of Mental Disorder in Relation to a Child's Specific Mental Disorder After the Child's Diagnosis in Finland......20

Supplement Figure 6. Men's Hazard Ratios and 95% Confidence Intervals of Mental Disorder in Relation to a Child's Specific Mental Disorder After the Child's Diagnosis in Finland......21

Description of Study Cohorts

Finland

Register data was constructed by linking the following registers: Care Register for Health Care (CRHC), the Hospital Discharge Register (HDR), and the Register of Primary Healthcare Visits (RPHV) maintained by the National Institute for Health and Welfare, and the full Finnish Population Register (FOLK) maintained by Statistics Finland. The CRHC and HDR contain information on public and private hospital admissions in Finland whereas the RPHV includes records of public and private primary healthcare visits in Finland. In the current study, HDR data from 1970 to 1993, CRHC data from 1994 onwards, and RPHV data from 2011 onwards were used. FOLK contains yearly updated administrative registers and includes basic demographic characteristics of the whole Finnish population.

Denmark

Register data was constructed by linking the following registers: the Central Person Register (CPR), the Danish Psychiatric Central Research Register, and the Danish Education Registers. The Central Person Register contains information on all people living in Denmark. It was established in 1968 and contains information including continually updated vital status. The Danish Psychiatric Central Research Register contains data on all psychiatric inpatient contacts since 1969, and all psychiatric outpatient and emergency contacts since 1995. The Danish Education Registers provide educational information on all individuals registered in Denmark from 1980 onwards.

Description of the Comorbidity Adjustment

Figure A shows an example situation where the follow-up begins 1/1/2010, and the parent is unexposed. A child receives an anxiety disorder diagnosis 15/2/2013, which is adjusted as comorbid anxiety from hereon. After that, a sibling receives a developmental disorder diagnosis, which is then adjusted as comorbid developmental disorder from hereon. 7/9/2016, the child gets a depressive disorder diagnosis, and the parent is exposed from hereon. Follow-up ends at administrative censoring, parent did not have the outcome. Children's diagnoses after exposure are not included in analyses (crossed out in the **Figure A)**.

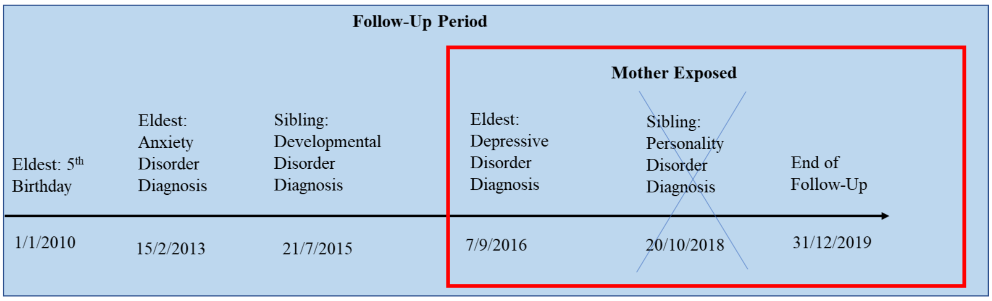

Figure A. Child's depressive disorder and parents's mental health: exposed mother

Another example situation is shown in **Figure B**; the follow-up begins 1/1/2010, the parent is unexposed. A child receives an anxiety disorder diagnosis 15/2/2013, which is adjusted as comorbid anxiety from hereon. After that, a sibling gets a developmental disorder diagnosis, which is then adjusted as comorbid developmental disorder from hereon. Sibling gets a personality disorder diagnosis, which is then adjusted as comorbid personality disorder from hereon. Follow-up ends at parent's anxiety diagnosis. Children's diagnoses after the end of follow-up are not included in analyses (crossed out in **Figure B**).

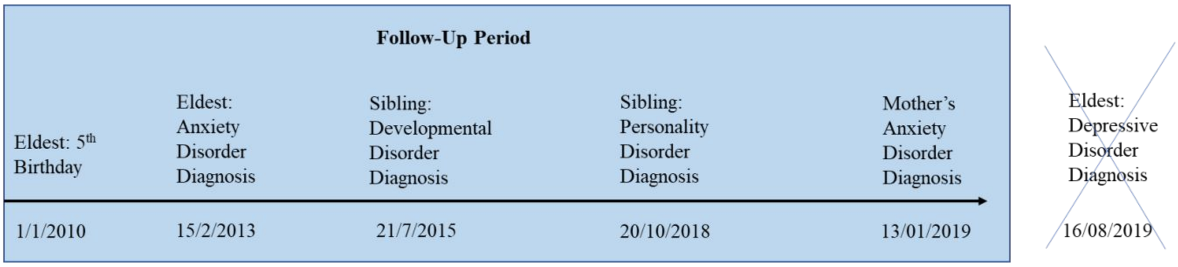

Figure B. Child's depressive disorder and mother's mental health: unexposed mother

Supplement Table 1. Diagnostic Categories of Mental Disorders in Finland and Denmark

Mental Disorder Category	ICD-10	Abbreviation	Finnish Conversions			Danish Conversions
			ICD-8 Equivalents	ICD-9 Equivalents	ICPC-2 Equivalents	ICD-8 Equivalents
Mental and behavioral disorders due to psychoactive substance use	F10–F19	Substance use disorders	291, 303, 304	291, 292, 303, 304, 305	P16, P15, P17, P19	291.x9, 294.39, 303.x9, 303.20, 303.28, 303.90, 304.x9
Schizophrenia, schizotypal and delusional disorders	F20–F29	Psychotic disorders	295, 297, 298.1, 298.2, 298.3, 298.9, 299	295, 297, 298	P72	295.x9, 296.89, 297.x9, 298.29–298.99, 299.04, 299.05, 299.09, 301.83
Mood (affective) disorders	F30–F39	Mood disorders	296, 298.0, 300.4, 301.1	296, 311	P73	296.x9 (Excluding 296.89), 298.09, 298.19, 300.49, 301.19
Neurotic, stress-related and somatoform disorders	F40–F48	Anxiety disorders	300.0, 300.1, 300.2, 300.3 300.5, 300.6, 300.7, 300.8, 300.9, 305, 306.8, 307, 308.4	300, 307.8, 309	P79, P74, P02, P82, P75, P78	300.x9 (Excluding 300.49), 305.x9, 305.68, 307.99
Eating Disorders	F50	Eating Disorders	305.60, 306.50, 306.58, 306.59	307.1, 307.5	P86	305.60, 306.50, 306.58, 306.59
Specific Personality Disorders	F60	Personality disorders	301.x9 (excluding	301	-	301.x9 (Excluding 301.19), 301.80, 301.81,

			301.19), 301.80, 301.81, 301.82, 301.84			301.82, 301.84
Intellectual disability	F70–F79	Intellectual disabilities	310, 311, 312, 313, 314, 315	317, 318, 319	P85	311.xx, 312.xx, 313.xx, 314.xx, 315.xx
Pervasive Developmental Disorders	F84	Developmental disorders	299.00, 299.01, 299.02, 299.03	299	-	299.00, 299.01, 299.02, 299.03
Behavioral and emotional disorders with onset in childhood and adolescence	F90–F98	Childhood onset disorders	306.2, 306.6, 306.7, 306.9, 308.1, 308.2 308.3	308.0, 307.2, 307.3, 307.6, 307.7, 307.9, 313, 314	P81, P22, P23, P10, P12, P13, P11	306.x9, 308.0x
Any mental disorder	F00–F99	Any mental disorder	All of the above + 290, 292, 293, 294, rest of 301, 302, 3060 3061, 3063, 3064, 3080, 309	All of the above + 290, 293, 294, 302, 3074, 312, 316, 310, 315	All of the above + P06, P07, P08, P09, P18, P24, P29, P70, P71, P76, P80, P98, P99	All of the above

Supplement Table 2. Women's Minimally and Comorbidity Adjusted Hazard Ratios and 95% Confidence Intervals for Mental Disorders in Relation to a Child's Specific Mental Disorder According to Time Since the Child's Diagnosis

Diagnosis	Model	Finland					Denmark				
		0-6m	6-12m	1-1.5y	1.5-2y	2y+	0-6m	6-12m	1-1.5y	1.5-2y	2y+
Any Mental Disorder	Minimally Adjusted	2.29 (2.21, 2.38)	1.83 (1.75, 1.91)	1.71 (1.64, 1.79)	1.66 (1.59, 1.74)	1.5 (1.48, 1.53)	2.54 (2.35, 2.75)	2.22 (2.04, 2.42)	1.92 (1.75, 2.11)	1.91 (1.74, 2.11)	1.77 (1.72, 1.83)
Child's Substance Use Disorder											
	Minimally Adjusted	2.07 (1.88, 2.28)	1.61 (1.43, 1.8)	1.63 (1.45, 1.83)	1.48 (1.31, 1.69)	1.33 (1.26, 1.4)	2.48 (1.88, 3.26)	2.01 (1.47, 2.76)	1.65 (1.16, 2.37)	1.87 (1.32, 2.65)	1.8 (1.59, 2.04)
	Comorbidity Adjusted	1.73 (1.57, 1.91)	1.36 (1.21, 1.52)	1.39 (1.24, 1.57)	1.29 (1.13, 1.46)	1.24 (1.18, 1.3)	1.81 (1.38, 2.39)	1.48 (1.08, 2.03)	1.23 (0.86, 1.76)	1.41 (1.00, 2.00)	1.43 (1.26, 1.62)
Child's Psychotic Disorder											
	Minimally Adjusted	3.71 (3.22, 4.28)	2.05 (1.69, 2.5)	1.86 (1.5, 2.31)	1.83 (1.46, 2.28)	1.51 (1.39, 1.65)	2.48 (1.92, 3.21)	1.76 (1.29, 2.41)	2.05 (1.52, 2.78)	1.78 (1.27, 2.49)	1.82 (1.62, 2.04)
	Comorbidity Adjusted	2.61 (2.27, 3.01)	1.46 (1.2, 1.78)	1.34 (1.08, 1.66)	1.33 (1.06, 1.66)	1.18 (1.08, 1.29)	1.76 (1.37, 2.28)	1.26 (0.92, 1.72)	1.47 (1.09, 1.99)	1.29 (0.92, 1.8)	1.38 (1.23, 1.55)
Child's Mood Disorder											
	Minimally Adjusted	2.73 (2.58, 2.88)	2.03 (1.89, 2.17)	1.71 (1.59, 1.85)	1.74 (1.61, 1.88)	1.6 (1.55, 1.65)	2.42 (2.03, 2.89)	2.32 (1.92, 2.79)	1.53 (1.2, 1.94)	1.74 (1.38, 2.19)	1.82 (1.68, 1.97)
	Comorbidity Adjusted	2.37 (2.24, 2.51)	1.77 (1.66, 1.89)	1.51 (1.4, 1.62)	1.54 (1.42, 1.66)	1.46 (1.42, 1.51)	2.01 (1.68, 2.39)	1.93 (1.6, 2.32)	1.28 (1.01, 1.62)	1.46 (1.16, 1.84)	1.6 (1.48, 1.73)
Child's Anxiety Disorder											
	Minimally Adjusted	2.31 (2.2, 2.43)	1.86 (1.75, 1.97)	1.55 (1.45, 1.66)	1.66 (1.55, 1.77)	1.51 (1.47, 1.55)	2.86 (2.57, 3.19)	2.1 (1.84, 2.39)	1.95 (1.7, 2.24)	1.82 (1.57, 2.11)	1.78 (1.69, 1.87)
	Comorbidity Adjusted	2.06 (1.96, 2.17)	1.66 (1.57, 1.76)	1.39 (1.3, 1.49)	1.49 (1.4, 1.6)	1.39 (1.35, 1.43)	2.54 (2.28, 2.82)	1.86 (1.64, 2.12)	1.74 (1.51, 1.99)	1.63 (1.41, 1.89)	1.63 (1.55, 1.72)
Child's Eating Disorder											
	Minimally Adjusted	2.32 (2.02, 2.67)	1.7 (1.44, 2.01)	1.62 (1.36, 1.93)	1.7 (1.43, 2.03)	1.28 (1.2, 1.37)	1.4 (0.98, 2.01)	1.59 (1.12, 2.24)	1.55 (1.07, 2.23)	1.52 (1.05, 2.22)	1.26 (1.1, 1.44)
	Comorbidity Adjusted	1.96 (1.7, 2.25)	1.45 (1.22, 1.71)	1.4 (1.17, 1.67)	1.5 (1.25, 1.79)	1.25 (1.17, 1.33)	1.26 (0.88, 1.8)	1.43 (1.01, 2.02)	1.41 (0.98, 2.02)	1.4 (0.96, 2.04)	1.24 (1.09, 1.42)
Child's Personality Disorder											
	Minimally Adjusted	1.89 (1.49, 2.4)	1.85 (1.44, 2.37)	1.55 (1.16, 2.07)	1.88 (1.43, 2.48)	1.57 (1.38, 1.78)	2.28 (1.74, 2.97)	2.08 (1.56, 2.77)	2.23 (1.68, 2.97)	1.72 (1.23, 2.41)	1.77 (1.58, 1.98)
	Comorbidity Adjusted	1.16 (0.91, 1.47)	1.14 (0.89, 1.47)	0.97 (0.72, 1.29)	1.18 (0.9, 1.56)	1.04 (0.91, 1.18)	1.58 (1.21, 2.06)	1.45 (1.09, 1.94)	1.57 (1.18, 2.09)	1.22 (0.87, 1.71)	1.32 (1.18, 1.49)
Child's Intellectual Disability											
	Minimally Adjusted	1.9 (1.56, 2.32)	1.43 (1.14, 1.79)	1.58 (1.27, 1.96)	1.52 (1.22, 1.9)	1.23 (1.16, 1.3)	2.06 (1.57, 2.72)	1.69 (1.25, 2.3)	1.41 (1, 1.98)	1.31 (0.91, 1.87)	1.53 (1.41, 1.67)
	Comorbidity Adjusted	1.63 (1.34, 1.99)	1.24 (0.99, 1.56)	1.38 (1.11, 1.72)	1.35 (1.08, 1.68)	1.24 (1.17, 1.31)	1.7 (1.29, 2.24)	1.41 (1.03, 1.91)	1.18 (0.84, 1.65)	1.1 (0.77, 1.57)	1.4 (1.29, 1.53)
Child's Developmental Disorder											
	Minimally Adjusted	2.82 (2.44, 3.26)	2.11 (1.78, 2.5)	1.89 (1.57, 2.27)	1.84 (1.52, 2.21)	1.43 (1.36, 1.5)	1.81 (1.52, 2.17)	2.14 (1.81, 2.53)	2.29 (1.94, 2.71)	1.93 (1.61, 2.33)	1.61 (1.52, 1.71)
	Comorbidity Adjusted	2.2 (1.9, 2.54)	1.67 (1.41, 1.97)	1.51 (1.25, 1.81)	1.48 (1.23, 1.79)	1.37 (1.3, 1.44)	1.54 (1.29, 1.85)	1.83 (1.55, 2.17)	1.98 (1.67, 2.33)	1.68 (1.39, 2.02)	1.52 (1.43, 1.61)

Diagnosis	Model	Finland					Denmark				
		0-6m	6-12m	1-1.5y	1.5-2y	2y+	0-6m	6-12m	1-1.5y	1.5-2y	2y+
Child's Childhood Onset Disorder											
	Minimally Adjusted	2.48 (2.35, 2.61)	1.93 (1.82, 2.05)	1.9 (1.79, 2.02)	1.72 (1.61, 1.83)	1.49 (1.46, 1.52)	2.62 (2.35, 2.91)	2.28 (2.03, 2.56)	2.22 (1.96, 2.5)	1.97 (1.73, 2.24)	1.72 (1.66, 1.8)
	Comorbidity Adjusted	2.33 (2.21, 2.45)	1.83 (1.72, 1.94)	1.81 (1.7, 1.92)	1.64 (1.54, 1.75)	1.51 (1.48, 1.54)	2.39 (2.15, 2.66)	2.09 (1.86, 2.35)	2.04 (1.81, 2.3)	1.82 (1.6, 2.08)	1.68 (1.62, 1.75)

Supplement Table 3. Men's Minimally and Comorbidity Adjusted Hazard Ratios and 95% Confidence Intervals for Mental Disorders in Relation to a Child’s Specific Mental Disorder According to Time Since the Child’s Diagnosis

Diagnosis	Model	Finland					Denmark				
		0-6m	6-12m	1-1.5y	1.5-2y	2y+	0-6m	6-12m	1-1.5y	1.5-2y	2y+
Any Mental Disorder	Minimally Adjusted	1.75 (1.67, 1.85)	1.58 (1.5, 1.67)	1.39 (1.31, 1.48)	1.4 (1.32, 1.49)	1.33 (1.3, 1.36)	2.04 (1.85, 2.24)	1.64 (1.47, 1.84)	1.92 (1.72, 2.13)	1.5 (1.33, 1.69)	1.51 (1.45, 1.56)
Child's Substance Use Disorder											
	Minimally Adjusted	1.72 (1.5, 1.97)	1.63 (1.41, 1.89)	1.43 (1.22, 1.68)	1.62 (1.39, 1.9)	1.37 (1.28, 1.46)	2.59 (1.89, 3.57)	1.01 (0.6, 1.7)	2.07 (1.42, 3.02)	2.36 (1.64, 3.39)	1.97 (1.72, 2.27)
	Comorbidity Adjusted	1.51 (1.32, 1.73)	1.45 (1.25, 1.67)	1.28 (1.09, 1.5)	1.46 (1.25, 1.71)	1.3 (1.22, 1.39)	2.05 (1.49, 2.81)	0.8 (0.47, 1.35)	1.65 (1.13, 2.41)	1.89 (1.31, 2.72)	1.64 (1.42, 1.89)
Child's Psychotic Disorder											
	Minimally Adjusted	2.04 (1.6, 2.59)	1.72 (1.32, 2.26)	2.1 (1.63, 2.71)	1.53 (1.13, 2.08)	1.58 (1.42, 1.75)	2.86 (2.17, 3.77)	1.68 (1.16, 2.44)	1.82 (1.25, 2.63)	1.95 (1.34, 2.82)	1.75 (1.53, 2.01)
	Comorbidity Adjusted	1.56 (1.22, 1.99)	1.33 (1.02, 1.74)	1.64 (1.27, 2.11)	1.2 (0.88, 1.63)	1.31 (1.18, 1.46)	2.19 (1.66, 2.89)	1.29 (0.89, 1.88)	1.4 (0.97, 2.04)	1.51 (1.04, 2.19)	1.42 (1.23, 1.63)
Child's Mood Disorder											
	Minimally Adjusted	1.78 (1.63, 1.94)	1.69 (1.54, 1.85)	1.4 (1.26, 1.55)	1.64 (1.49, 1.81)	1.44 (1.39, 1.5)	2.12 (1.72, 2.62)	1.79 (1.41, 2.27)	1.97 (1.55, 2.49)	1.3 (0.96, 1.75)	1.63 (1.48, 1.78)
	Comorbidity Adjusted	1.6 (1.46, 1.74)	1.52 (1.38, 1.66)	1.26 (1.14, 1.4)	1.49 (1.35, 1.65)	1.35 (1.3, 1.41)	1.85 (1.49, 2.29)	1.56 (1.23, 1.98)	1.72 (1.36, 2.19)	1.14 (0.85, 1.54)	1.48 (1.35, 1.63)
Child's Anxiety Disorder											
	Minimally Adjusted	1.81 (1.68, 1.94)	1.57 (1.45, 1.7)	1.47 (1.35, 1.6)	1.39 (1.27, 1.52)	1.4 (1.35, 1.45)	2.16 (1.88, 2.49)	1.72 (1.46, 2.02)	1.73 (1.46, 2.04)	1.28 (1.05, 1.55)	1.59 (1.5, 1.69)
	Comorbidity Adjusted	1.66 (1.54, 1.78)	1.44 (1.33, 1.56)	1.36 (1.25, 1.48)	1.29 (1.18, 1.41)	1.32 (1.27, 1.36)	1.98 (1.72, 2.27)	1.57 (1.34, 1.85)	1.58 (1.34, 1.87)	1.17 (0.96, 1.43)	1.49 (1.41, 1.59)
Child's Eating Disorder											
	Minimally Adjusted	1.61 (1.3, 1.99)	1.51 (1.2, 1.89)	1.19 (0.92, 1.55)	1.4 (1.09, 1.78)	1.17 (1.07, 1.27)	1.79 (1.25, 2.56)	1.26 (0.82, 1.96)	1.98 (1.37, 2.85)	1.08 (0.65, 1.8)	1.26 (1.09, 1.47)
	Comorbidity Adjusted	1.42 (1.14, 1.75)	1.34 (1.07, 1.68)	1.07 (0.83, 1.39)	1.27 (0.99, 1.62)	1.15 (1.06, 1.25)	1.65 (1.15, 2.36)	1.17 (0.76, 1.82)	1.85 (1.28, 2.66)	1.02 (0.62, 1.69)	1.25 (1.08, 1.46)
Child's Personality Disorder											
	Minimally Adjusted	1.41 (1, 1.98)	2.23 (1.68, 2.96)	1.55 (1.08, 2.22)	1.87 (1.33, 2.64)	1.21 (1.02, 1.44)	1.89 (1.35, 2.65)	1.82 (1.28, 2.58)	2.14 (1.53, 2.99)	1.34 (0.86, 2.07)	1.51 (1.31, 1.74)
	Comorbidity Adjusted	0.97 (0.69, 1.37)	1.55 (1.17, 2.06)	1.08 (0.76, 1.55)	1.31 (0.93, 1.85)	0.89 (0.75, 1.06)	1.38 (0.99, 1.94)	1.33 (0.94, 1.9)	1.58 (1.13, 2.21)	0.99 (0.64, 1.54)	1.18 (1.02, 1.36)
Child's Intellectual Disability											
	Minimally Adjusted	1.65 (1.26, 2.14)	1.67 (1.29, 2.17)	1.39 (1.04, 1.86)	1.03 (0.74, 1.44)	1.25 (1.16, 1.34)	1.58 (1.09, 2.29)	2.07 (1.49, 2.87)	1.54 (1.05, 2.26)	1.51 (1.02, 2.24)	1.48 (1.33, 1.63)
	Comorbidity Adjusted	1.48 (1.14, 1.93)	1.51 (1.16, 1.97)	1.27 (0.95, 1.7)	0.95 (0.68, 1.33)	1.27 (1.18, 1.36)	1.38 (0.96, 2.01)	1.82 (1.31, 2.52)	1.36 (0.93, 2)	1.34 (0.91, 1.99)	1.4 (1.26, 1.55)
Child's Developmental Disorder											
	Minimally Adjusted	1.65 (1.31, 2.08)	1.25 (0.96, 1.63)	1.78 (1.42, 2.24)	1.52 (1.19, 1.95)	1.36 (1.28, 1.45)	1.82 (1.49, 2.23)	1.35 (1.06, 1.71)	1.51 (1.2, 1.9)	1.6 (1.28, 2.01)	1.27 (1.18, 1.36)
	Comorbidity Adjusted	1.39 (1.1, 1.75)	1.06 (0.81, 1.39)	1.52 (1.21, 1.92)	1.32 (1.03, 1.69)	1.32 (1.24, 1.4)	1.61 (1.31, 1.97)	1.19 (0.94, 1.52)	1.35 (1.07, 1.7)	1.44 (1.14, 1.81)	1.21 (1.13, 1.3)
Child's Childhood Onset Disorder											

Diagnosis	Model	Finland					Denmark				
		0-6m	6-12m	1-1.5y	1.5-2y	2y+	0-6m	6-12m	1-1.5y	1.5-2y	2y+
	Minimally Adjusted	1.8 (1.67, 1.94)	1.71 (1.59, 1.85)	1.52 (1.4, 1.65)	1.49 (1.37, 1.62)	1.34 (1.3, 1.37)	2.15 (1.88, 2.46)	1.84 (1.59, 2.13)	2.01 (1.74, 2.33)	1.84 (1.58, 2.14)	1.46 (1.39, 1.53)
	Comorbidity Adjusted	1.72 (1.6, 1.85)	1.65 (1.52, 1.78)	1.47 (1.35, 1.6)	1.45 (1.33, 1.57)	1.35 (1.32, 1.39)	2.02 (1.76, 2.31)	1.73 (1.49, 2.01)	1.9 (1.65, 2.2)	1.75 (1.5, 2.03)	1.44 (1.37, 1.51)

Supplement Table 4. Frequencies of Persons Diagnosed Prior to Follow-up, at Risk at Start of Follow-up, Diagnosed or Otherwise Censored During Follow-up, and Exposed During Follow-up in the Sensitivity Analyses.

		Finland		Denmark	
		Women	Men	Women	Men
Mental Disorder Diagnosis Prior to Follow-up					
	Early Follow-up Start	17893	21768	17883	12141
	Late Follow-up Start	28696	29331	27641	18625
Persons at Risk at Start of Follow-up					
	Early Follow-up Start	394700	381683	444013	441125
	Late Follow-up Start	381476	371345	425104	424032
New Cases During Follow-up					
	Early Follow-up Start	107203	68787	54007	40705
	Late Follow-up Start	96400	61224	44249	34221
Emigrated During Follow-up					
	Early Follow-up Start	7823	7735	24267	29914
	Late Follow-up Start	5693	5986	15495	20316
Died During Follow-up					
	Early Follow-up Start	3167	8098	6206	11797
	Late Follow-up Start	2876	7072	5827	10786
Exposed During Follow-up					
	Any Mental Disorder	160904	160752	97233	97848
	Substance Use Disorders	17585	17453	6996	6818
	Psychotic Disorders	4995	5088	9184	9050
	Mood Disorders	45697	46877	21426	21426
	Anxiety Disorders	66856	68420	45093	45648
	Eating Disorders	8846	8969	8963	8932
	Personality Disorders	3194	3379	8557	8606
	Intellectual Disabilities	6366	6429	6685	6702
	Developmental Disorders	8288	8573	22418	22892
	Childhood Onset Disorders	67974	69553	40533	41307

Supplement Table 5. Descriptives of Study Population in Sensitivity Analyses.

	Finland				Denmark			
	Women		Men		Women		Men	
	Follow-up Started at 1	Follow-up Started at 5	Follow-up Started at 1	Follow-up Started at 5	Follow-up Started at 1	Follow-up Started at 5	Follow-up Started at 1	Follow-up Started at 5
	Median (IQR)	*Median (IQR)*	*Median (IQR)*	*Median (IQR)*	*Median (IQR)*	*Median (IQR)*	*Median (IQR)*	*Median (IQR)*
Parent's Birth Year, *N (%)*								
<1950	735 (0.2%)	726 (0.2%)	4087 (1.1%)	3964 (1.1%)	387 (<0.1%)	370 (<0.1%)	4262 (1.0%)	4043 (1.0%)
1951-1960	29677 (7.5%)	29086 (7.6%)	50925 (13%)	49969 (13%)	26339 (5.9%)	25378 (6.0%)	54635 (12%)	52760 (12%)
1961-1970	188805 (48%)	184661 (48%)	195078 (51%)	191283 (52%)	215224 (48%)	207893 (49%)	232405 (53%)	224633 (53%)
1971-1980	152577 (39%)	146342 (38%)	116492 (31%)	112384 (30%)	183941 (41%)	175285 (41%)	139955 (32%)	133762 (32%)
>1980	22906 (5.8%)	20661 (5.4%)	15101 (4.0%)	13745 (3.7%)	18122 (4.1%)	16178 (3.8%)	9868 (2.2%)	8834 (2.1%)
Parent's Age at Start of Follow-up	28 (24, 31)	32 (28, 35)	29 (26, 33)	33 (30, 37)	27 (25, 30)	31 (29, 34)	29 (26, 33)	33 (30, 37)
Parent's Age at End of Follow-up	47 (42, 53)	48 (42, 53)	50 (44, 55)	50 (45, 55)	50 (44, 55)	50 (45, 55)	52 (46, 57)	52 (47, 57)
Parent's Education at Start of Follow-up, *N (%)*								
Lower secondary or less	58007 (15%)	46791 (12%)	71952 (19%)	61495 (17%)	96402 (22%)	83887 (20%)	96529 (22%)	85948 (20%)
Upper secondary	157774 (40%)	146351 (38%)	184977 (48%)	173620 (47%)	227832 (52%)	214542 (51%)	252070 (58%)	242285 (58%)
Post-secondary or tertiary	178919 (45%)	188334 (49%)	124754 (33%)	136230 (37%)	112635 (26%)	123802 (29%)	84239 (19%)	91895 (22%)
Child's Age at Substance Use Disorder Diagnosis		17 (15, 20)		17 (15, 20)		19 (17, 22)		19 (17, 22)
Child's Age at Psychotic Disorder Diagnosis		17 (15, 20)		17 (15, 20)		19 (16, 21)		18 (16, 21)
Child's Age at Mood Disorder Diagnosis		16 (14, 19)		16 (14, 19)		18 (16, 21)		18 (16, 21)
Child's Age at Anxiety Disorder Diagnosis		16 (13, 19)		16 (13, 19)		16 (14, 19)		16 (14, 19)
Child's Age at Eating Disorder Diagnosis		15 (13, 17)		15 (13, 17)		16 (14, 19)		16 (14, 19)
Child's Age at Personality Disorder Diagnosis		19 (18, 21)		19 (18, 21)		19 (17, 22)		19 (17, 22)
Child's Age at Intellectual Disability Diagnosis	6 (4, 12)		6 (4, 12)		11 (6, 16)		12 (7, 16)	
Child's Age at Developmental Disorder Diagnosis	8 (5, 12)		8 (5, 12)		12 (8, 15)		12 (8, 15)	
Child's Age at Childhood Onset Disorder Diagnosis	8 (5, 12)		8 (5, 12)		12 (8, 15)		12 (8, 15)	
Child's Age at First Mental Disorder Diagnosis	11 (5, 16)		11 (5, 16)		15 (10, 18)		15 (10, 18)	

Supplement Table 6. Women's Minimally and Comorbidity Adjusted Hazard Ratios and 95% Confidence Intervals for Mental Disorders in Relation to a Child’s Specific Mental Disorder Before and After the Child’s Diagnosis

Diagnosis	Model	Finland							Denmark						
		Before		After					Before		After				
		12-6m	6-0m	0-6m	6-12m	1-1.5y	1.5-2y	2y+	12-6m	6-0m	0-6m	6-12m	1-1.5y	1.5-2y	2y+
Any Mental Disorder	Minimally Adjusted	1.8 (1.73, 1.88)	2.26 (2.18, 2.35)	2.42 (2.33, 2.52)	1.91 (1.83, 1.99)	1.8 (1.72, 1.88)	1.74 (1.66, 1.82)	1.58 (1.55, 1.6)	1.99 (1.83, 2.17)	2.65 (2.45, 2.85)	2.76 (2.56, 2.98)	2.29 (2.11, 2.5)	1.99 (1.82, 2.19)	1.97 (1.79, 2.17)	1.84 (1.78, 1.9)
Child's Substance Use Disorder															
	Minimally Adjusted	1.66 (1.49, 1.84)	2.03 (1.85, 2.24)	2.09 (1.9, 2.31)	1.61 (1.44, 1.81)	1.65 (1.47, 1.86)	1.48 (1.3, 1.68)	1.34 (1.27, 1.41)	2.29 (1.74, 3)	1.87 (1.38, 2.55)	2.43 (1.84, 3.21)	2.07 (1.51, 2.82)	1.65 (1.15, 2.35)	1.88 (1.33, 2.67)	1.8 (1.59, 2.04)
	Comorbidity Adjusted	1.35 (1.21, 1.51)	1.68 (1.53, 1.85)	1.75 (1.59, 1.93)	1.37 (1.22, 1.53)	1.42 (1.27, 1.6)	1.29 (1.14, 1.47)	1.26 (1.2, 1.33)	1.62 (1.24, 2.13)	1.34 (0.98, 1.82)	1.75 (1.33, 2.31)	1.5 (1.1, 2.05)	1.21 (0.84, 1.73)	1.4 (0.99, 1.98)	1.41 (1.25, 1.6)
Child's Psychotic Disorder															
	Minimally Adjusted	1.58 (1.28, 1.96)	2.38 (2, 2.83)	3.76 (3.26, 4.33)	2.06 (1.69, 2.5)	1.86 (1.5, 2.3)	1.84 (1.47, 2.3)	1.51 (1.39, 1.65)	2.05 (1.58, 2.68)	2.26 (1.75, 2.93)	2.49 (1.93, 3.21)	1.76 (1.29, 2.41)	2.04 (1.51, 2.77)	1.74 (1.23, 2.45)	1.83 (1.62, 2.05)
	Comorbidity Adjusted	1.06 (0.86, 1.31)	1.62 (1.36, 1.93)	2.58 (2.24, 2.98)	1.43 (1.18, 1.75)	1.31 (1.06, 1.62)	1.31 (1.05, 1.64)	1.18 (1.08, 1.28)	1.41 (1.08, 1.84)	1.57 (1.21, 2.03)	1.73 (1.34, 2.24)	1.23 (0.9, 1.69)	1.44 (1.06, 1.95)	1.23 (0.88, 1.74)	1.37 (1.21, 1.54)
Child's Mood Disorder															
	Minimally Adjusted	1.81 (1.7, 1.94)	2.43 (2.29, 2.57)	2.81 (2.65, 2.97)	2.07 (1.93, 2.21)	1.76 (1.63, 1.9)	1.78 (1.65, 1.93)	1.64 (1.59, 1.69)	2.16 (1.81, 2.57)	2.41 (2.03, 2.86)	2.42 (2.03, 2.89)	2.33 (1.94, 2.81)	1.55 (1.22, 1.96)	1.74 (1.38, 2.19)	1.83 (1.69, 1.98)
	Comorbidity Adjusted	1.54 (1.43, 1.64)	2.07 (1.95, 2.2)	2.41 (2.28, 2.55)	1.79 (1.67, 1.91)	1.53 (1.42, 1.65)	1.56 (1.44, 1.68)	1.49 (1.45, 1.54)	1.74 (1.46, 2.08)	1.96 (1.65, 2.33)	1.98 (1.66, 2.37)	1.92 (1.59, 2.31)	1.28 (1.01, 1.63)	1.44 (1.15, 1.82)	1.6 (1.48, 1.74)
Child's Anxiety Disorder															
	Minimally Adjusted	1.85 (1.75, 1.96)	2.48 (2.36, 2.6)	2.45 (2.33, 2.57)	1.92 (1.81, 2.03)	1.61 (1.5, 1.72)	1.72 (1.61, 1.84)	1.57 (1.52, 1.61)	1.95 (1.72, 2.21)	3.37 (3.06, 3.71)	3.24 (2.92, 3.58)	2.16 (1.9, 2.45)	1.99 (1.74, 2.29)	1.84 (1.58, 2.13)	1.81 (1.72, 1.91)
	Comorbidity Adjusted	1.64 (1.55, 1.74)	2.21 (2.1, 2.32)	2.18 (2.08, 2.3)	1.72 (1.62, 1.82)	1.44 (1.35, 1.54)	1.55 (1.45, 1.66)	1.44 (1.4, 1.49)	1.72 (1.52, 1.95)	2.99 (2.72, 3.3)	2.88 (2.6, 3.19)	1.92 (1.69, 2.19)	1.79 (1.56, 2.05)	1.65 (1.42, 1.91)	1.67 (1.59, 1.76)
Child's Eating Disorder															
	Minimally Adjusted	1.39 (1.16, 1.67)	1.89 (1.62, 2.21)	2.33 (2.02, 2.68)	1.72 (1.45, 2.03)	1.63 (1.37, 1.94)	1.69 (1.42, 2.02)	1.28 (1.2, 1.37)	1.56 (1.12, 2.16)	1.38 (0.97, 1.96)	1.5 (1.06, 2.12)	1.59 (1.12, 2.25)	1.54 (1.07, 2.22)	1.54 (1.05, 2.24)	1.26 (1.11, 1.45)
	Comorbidity Adjusted	1.14 (0.95, 1.37)	1.58 (1.35, 1.84)	1.96 (1.71, 2.26)	1.47 (1.24, 1.73)	1.42 (1.19, 1.69)	1.5 (1.25, 1.79)	1.28 (1.2, 1.37)	1.37 (0.99, 1.9)	1.22 (0.86, 1.73)	1.34 (0.95, 1.9)	1.43 (1.01, 2.03)	1.4 (0.98, 2.02)	1.42 (0.97, 2.07)	1.26 (1.1, 1.44)
Child's Personality Disorder															
	Minimally Adjusted	1.91 (1.52, 2.41)	1.92 (1.53, 2.42)	1.89 (1.49, 2.39)	1.85 (1.44, 2.37)	1.51 (1.12, 2.02)	1.89 (1.44, 2.49)	1.57 (1.38, 1.78)	2 (1.52, 2.62)	2.67 (2.1, 3.39)	2.29 (1.75, 2.99)	2.09 (1.57, 2.78)	2.23 (1.68, 2.97)	1.74 (1.24, 2.44)	1.77 (1.58, 1.98)

Diagnosis	Model	Finland							Denmark						
		Before		After					Before		After				
		12-6m	6-0m	0-6m	6-12m	1-1.5y	1.5-2y	2y+	12-6m	6-0m	0-6m	6-12m	1-1.5y	1.5-2y	2y+
	Comorbidity Adjusted	1.11 (0.88, 1.4)	1.13 (0.9, 1.42)	1.12 (0.89, 1.43)	1.11 (0.86, 1.43)	0.89 (0.66, 1.2)	1.16 (0.88, 1.53)	1.02 (0.89, 1.15)	1.33 (1.01, 1.75)	1.79 (1.41, 2.28)	1.55 (1.18, 2.02)	1.42 (1.07, 1.9)	1.53 (1.15, 2.04)	1.21 (0.86, 1.69)	1.3 (1.16, 1.46)
Child's Intellectual Disability	Minimally Adjusted	1.91 (1.56, 2.34)	1.57 (1.26, 1.96)	1.89 (1.55, 2.31)	1.42 (1.13, 1.78)	1.6 (1.29, 1.98)	1.54 (1.23, 1.92)	1.23 (1.17, 1.3)	1.5 (1.09, 2.06)	2.12 (1.62, 2.77)	2.05 (1.56, 2.71)	1.68 (1.23, 2.29)	1.42 (1.01, 2)	1.33 (0.93, 1.91)	1.55 (1.42, 1.69)
	Comorbidity Adjusted	1.61 (1.31, 1.97)	1.32 (1.06, 1.65)	1.63 (1.34, 1.99)	1.24 (0.98, 1.55)	1.41 (1.14, 1.75)	1.38 (1.1, 1.72)	1.29 (1.22, 1.36)	1.21 (0.88, 1.66)	1.72 (1.31, 2.25)	1.69 (1.28, 2.23)	1.39 (1.02, 1.89)	1.19 (0.84, 1.67)	1.12 (0.78, 1.6)	1.43 (1.32, 1.56)
Child's Developmental Disorder	Minimally Adjusted	2.19 (1.85, 2.58)	2.3 (1.96, 2.7)	2.82 (2.44, 3.26)	2.11 (1.78, 2.5)	1.9 (1.58, 2.28)	1.85 (1.54, 2.23)	1.43 (1.36, 1.5)	1.75 (1.46, 2.09)	1.79 (1.5, 2.13)	1.81 (1.51, 2.16)	2.13 (1.8, 2.52)	2.29 (1.94, 2.7)	1.95 (1.62, 2.35)	1.61 (1.52, 1.71)
	Comorbidity Adjusted	1.64 (1.39, 1.93)	1.74 (1.48, 2.04)	2.15 (1.86, 2.49)	1.64 (1.38, 1.94)	1.5 (1.25, 1.8)	1.49 (1.23, 1.79)	1.39 (1.33, 1.46)	1.45 (1.21, 1.73)	1.49 (1.25, 1.78)	1.52 (1.27, 1.82)	1.81 (1.52, 2.14)	1.96 (1.66, 2.31)	1.68 (1.4, 2.02)	1.53 (1.45, 1.62)
Child's Childhood Onset Disorder	Minimally Adjusted	2.1 (1.99, 2.23)	2.56 (2.43, 2.7)	2.57 (2.44, 2.71)	1.98 (1.87, 2.1)	1.95 (1.84, 2.07)	1.76 (1.65, 1.88)	1.52 (1.49, 1.55)	2.29 (2.05, 2.56)	2.21 (1.97, 2.48)	2.66 (2.39, 2.96)	2.33 (2.07, 2.62)	2.25 (2, 2.54)	2.01 (1.76, 2.29)	1.75 (1.68, 1.83)
	Comorbidity Adjusted	1.96 (1.85, 2.07)	2.4 (2.27, 2.52)	2.42 (2.3, 2.55)	1.88 (1.77, 1.99)	1.86 (1.76, 1.98)	1.69 (1.59, 1.8)	1.57 (1.54, 1.61)	2.05 (1.84, 2.3)	1.99 (1.77, 2.23)	2.41 (2.17, 2.68)	2.13 (1.89, 2.39)	2.07 (1.83, 2.33)	1.85 (1.63, 2.11)	1.73 (1.66, 1.8)

Supplement Table 7. Men's Minimally and Comorbidity Adjusted Hazard Ratios and 95% Confidence Intervals for Mental Disorders in Relation to a Child's Specific Mental Disorder Before and After the Child's Diagnosis

Diagnosis	Model	Finland							Denmark						
		Before		After					Before		After				
		12-6m	6-0m	0-6m	6-12m	1-1.5y	1.5-2y	2y+	12-6m	6-0m	0-6m	6-12m	1-1.5y	1.5-2y	2y+
Any Mental Disorder	Minimally Adjusted	1.7 (1.61, 1.79)	1.82 (1.72, 1.91)	1.82 (1.72, 1.91)	1.64 (1.55, 1.73)	1.43 (1.35, 1.52)	1.45 (1.37, 1.54)	1.37 (1.35, 1.4)	1.68 (1.52, 1.87)	2.21 (2.02, 2.43)	2.14 (1.95, 2.36)	1.68 (1.5, 1.87)	1.94 (1.74, 2.15)	1.53 (1.35, 1.72)	1.54 (1.48, 1.6)
Child's Substance Use Disorder															
	Minimally Adjusted	1.66 (1.49, 1.84)	2.03 (1.85, 2.24)	2.09 (1.9, 2.31)	1.61 (1.44, 1.81)	1.65 (1.47, 1.86)	1.48 (1.3, 1.68)	1.34 (1.27, 1.41)	2.29 (1.74, 3)	1.87 (1.38, 2.55)	2.43 (1.84, 3.21)	2.07 (1.51, 2.82)	1.65 (1.15, 2.35)	1.88 (1.33, 2.67)	1.8 (1.59, 2.04)
	Comorbidity Adjusted	1.35 (1.21, 1.51)	1.68 (1.53, 1.85)	1.75 (1.59, 1.93)	1.37 (1.22, 1.53)	1.42 (1.27, 1.6)	1.29 (1.14, 1.47)	1.26 (1.2, 1.33)	1.62 (1.24, 2.13)	1.34 (0.98, 1.82)	1.75 (1.33, 2.31)	1.5 (1.1, 2.05)	1.21 (0.84, 1.73)	1.4 (0.99, 1.98)	1.41 (1.25, 1.6)
Child's Psychotic Disorder															
	Minimally Adjusted	1.58 (1.28, 1.96)	2.38 (2, 2.83)	3.76 (3.26, 4.33)	2.06 (1.69, 2.5)	1.86 (1.5, 2.3)	1.84 (1.47, 2.3)	1.51 (1.39, 1.65)	2.05 (1.58, 2.68)	2.26 (1.75, 2.93)	2.49 (1.93, 3.21)	1.76 (1.29, 2.41)	2.04 (1.51, 2.77)	1.74 (1.23, 2.45)	1.83 (1.62, 2.05)
	Comorbidity Adjusted	1.06 (0.86, 1.31)	1.62 (1.36, 1.93)	2.58 (2.24, 2.98)	1.43 (1.18, 1.75)	1.31 (1.06, 1.62)	1.31 (1.05, 1.64)	1.18 (1.08, 1.28)	1.41 (1.08, 1.84)	1.57 (1.21, 2.03)	1.73 (1.34, 2.24)	1.23 (0.9, 1.69)	1.44 (1.06, 1.95)	1.23 (0.88, 1.74)	1.37 (1.21, 1.54)
Child's Mood Disorder															
	Minimally Adjusted	1.81 (1.7, 1.94)	2.43 (2.29, 2.57)	2.81 (2.65, 2.97)	2.07 (1.93, 2.21)	1.76 (1.63, 1.9)	1.78 (1.65, 1.93)	1.64 (1.59, 1.69)	2.16 (1.81, 2.57)	2.41 (2.03, 2.86)	2.42 (2.03, 2.89)	2.33 (1.94, 2.81)	1.55 (1.22, 1.96)	1.74 (1.38, 2.19)	1.83 (1.69, 1.98)
	Comorbidity Adjusted	1.54 (1.43, 1.64)	2.07 (1.95, 2.2)	2.41 (2.28, 2.55)	1.79 (1.67, 1.91)	1.53 (1.42, 1.65)	1.56 (1.44, 1.68)	1.49 (1.45, 1.54)	1.74 (1.46, 2.08)	1.96 (1.65, 2.33)	1.98 (1.66, 2.37)	1.92 (1.59, 2.31)	1.28 (1.01, 1.63)	1.44 (1.15, 1.82)	1.6 (1.48, 1.74)
Child's Anxiety Disorder															
	Minimally Adjusted	1.85 (1.75, 1.96)	2.48 (2.36, 2.6)	2.45 (2.33, 2.57)	1.92 (1.81, 2.03)	1.61 (1.5, 1.72)	1.72 (1.61, 1.84)	1.57 (1.52, 1.61)	1.95 (1.72, 2.21)	3.37 (3.06, 3.71)	3.24 (2.92, 3.58)	2.16 (1.9, 2.45)	1.99 (1.74, 2.29)	1.84 (1.58, 2.13)	1.81 (1.72, 1.91)
	Comorbidity Adjusted	1.64 (1.55, 1.74)	2.21 (2.1, 2.32)	2.18 (2.08, 2.3)	1.72 (1.62, 1.82)	1.44 (1.35, 1.54)	1.55 (1.45, 1.66)	1.44 (1.4, 1.49)	1.72 (1.52, 1.95)	2.99 (2.72, 3.3)	2.88 (2.6, 3.19)	1.92 (1.69, 2.19)	1.79 (1.56, 2.05)	1.65 (1.42, 1.91)	1.67 (1.59, 1.76)
Child's Eating Disorder															
	Minimally Adjusted	1.39 (1.16, 1.67)	1.89 (1.62, 2.21)	2.33 (2.02, 2.68)	1.72 (1.45, 2.03)	1.63 (1.37, 1.94)	1.69 (1.42, 2.02)	1.28 (1.2, 1.37)	1.56 (1.12, 2.16)	1.38 (0.97, 1.96)	1.5 (1.06, 2.12)	1.59 (1.12, 2.25)	1.54 (1.07, 2.22)	1.54 (1.05, 2.24)	1.26 (1.11, 1.45)
	Comorbidity Adjusted	1.14 (0.95, 1.37)	1.58 (1.35, 1.84)	1.96 (1.71, 2.26)	1.47 (1.24, 1.73)	1.42 (1.19, 1.69)	1.5 (1.25, 1.79)	1.28 (1.2, 1.37)	1.37 (0.99, 1.9)	1.22 (0.86, 1.73)	1.34 (0.95, 1.9)	1.43 (1.01, 2.03)	1.4 (0.98, 2.02)	1.42 (0.97, 2.07)	1.26 (1.1, 1.44)
Child's Personality Disorder															
	Minimally Adjusted	1.91 (1.52, 2.41)	1.92 (1.53, 2.42)	1.89 (1.49, 2.39)	1.85 (1.44, 2.37)	1.51 (1.12, 2.02)	1.89 (1.44, 2.49)	1.57 (1.38, 1.78)	2 (1.52, 2.62)	2.67 (2.1, 3.39)	2.29 (1.75, 2.99)	2.09 (1.57, 2.78)	2.23 (1.68, 2.97)	1.74 (1.24, 2.44)	1.77 (1.58, 1.98)
	Comorbidity Adjusted	1.11 (0.88, 1.4)	1.13 (0.9, 1.42)	1.12 (0.89, 1.43)	1.11 (0.86, 1.43)	0.89 (0.66, 1.2)	1.16 (0.88, 1.53)	1.02 (0.89, 1.15)	1.33 (1.01, 1.75)	1.79 (1.41, 2.28)	1.55 (1.18, 2.02)	1.42 (1.07, 1.9)	1.53 (1.15, 2.04)	1.21 (0.86, 1.69)	1.3 (1.16, 1.46)
Child's Intellectual Disability															

Diagnosis	Model	Finland							Denmark						
		Before		After					Before		After				
		12-6m	6-0m	0-6m	6-12m	1-1.5y	1.5-2y	2y+	12-6m	6-0m	0-6m	6-12m	1-1.5y	1.5-2y	2y+
	Minimally Adjusted	1.91 (1.56, 2.34)	1.57 (1.26, 1.96)	1.89 (1.55, 2.31)	1.42 (1.13, 1.78)	1.6 (1.29, 1.98)	1.54 (1.23, 1.92)	1.23 (1.17, 1.3)	1.5 (1.09, 2.06)	2.12 (1.62, 2.77)	2.05 (1.56, 2.71)	1.68 (1.23, 2.29)	1.42 (1.01, 2)	1.33 (0.93, 1.91)	1.55 (1.42, 1.69)
	Comorbidity Adjusted	1.61 (1.31, 1.97)	1.32 (1.06, 1.65)	1.63 (1.34, 1.99)	1.24 (0.98, 1.55)	1.41 (1.14, 1.75)	1.38 (1.1, 1.72)	1.29 (1.22, 1.36)	1.21 (0.88, 1.66)	1.72 (1.31, 2.25)	1.69 (1.28, 2.23)	1.39 (1.02, 1.89)	1.19 (0.84, 1.67)	1.12 (0.78, 1.6)	1.43 (1.32, 1.56)
Child's Developmental Disorder															
	Minimally Adjusted	2.19 (1.85, 2.58)	2.3 (1.96, 2.7)	2.82 (2.44, 3.26)	2.11 (1.78, 2.5)	1.9 (1.58, 2.28)	1.85 (1.54, 2.23)	1.43 (1.36, 1.5)	1.75 (1.46, 2.09)	1.79 (1.5, 2.13)	1.81 (1.51, 2.16)	2.13 (1.8, 2.52)	2.29 (1.94, 2.7)	1.95 (1.62, 2.35)	1.61 (1.52, 1.71)
	Comorbidity Adjusted	1.64 (1.39, 1.93)	1.74 (1.48, 2.04)	2.15 (1.86, 2.49)	1.64 (1.38, 1.94)	1.5 (1.25, 1.8)	1.49 (1.23, 1.79)	1.39 (1.33, 1.46)	1.45 (1.21, 1.73)	1.49 (1.25, 1.78)	1.52 (1.27, 1.82)	1.81 (1.52, 2.14)	1.96 (1.66, 2.31)	1.68 (1.4, 2.02)	1.53 (1.45, 1.62)
Child's Childhood Onset Disorder															
	Minimally Adjusted	2.1 (1.99, 2.23)	2.56 (2.43, 2.7)	2.57 (2.44, 2.71)	1.98 (1.87, 2.1)	1.95 (1.84, 2.07)	1.76 (1.65, 1.88)	1.52 (1.49, 1.55)	2.29 (2.05, 2.56)	2.21 (1.97, 2.48)	2.66 (2.39, 2.96)	2.33 (2.07, 2.62)	2.25 (2, 2.54)	2.01 (1.76, 2.29)	1.75 (1.68, 1.83)
	Comorbidity Adjusted	1.96 (1.85, 2.07)	2.4 (2.27, 2.52)	2.42 (2.3, 2.55)	1.88 (1.77, 1.99)	1.86 (1.76, 1.98)	1.69 (1.59, 1.8)	1.57 (1.54, 1.61)	2.05 (1.84, 2.3)	1.99 (1.77, 2.23)	2.41 (2.17, 2.68)	2.13 (1.89, 2.39)	2.07 (1.83, 2.33)	1.85 (1.63, 2.11)	1.73 (1.66, 1.8)

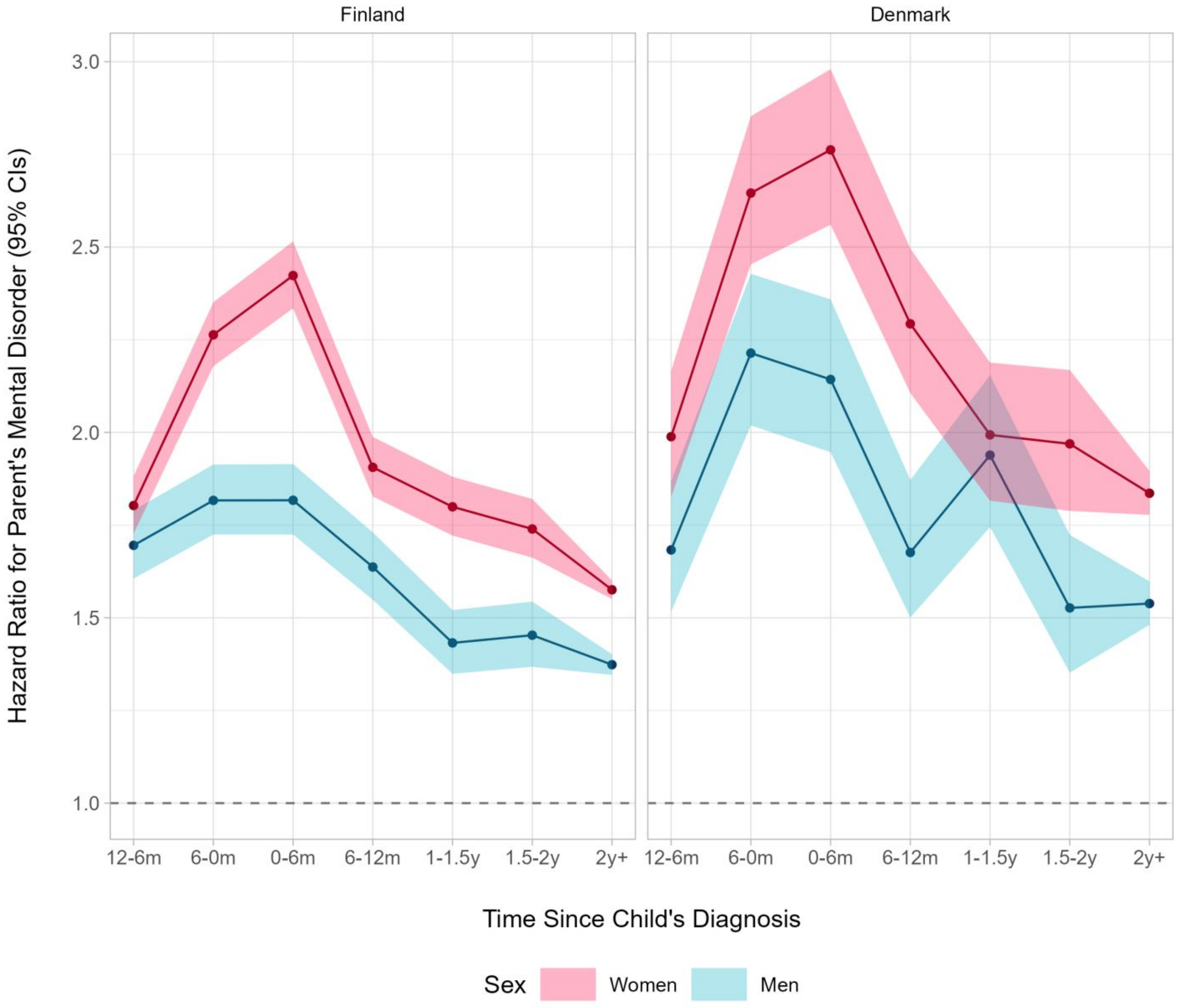

Supplement Figure 1. Hazard Ratios and 95% Confidence Intervals of Parents' Mental Disorder in Relation to Any Mental Disorder of a Child Before and After the Child's Diagnosis

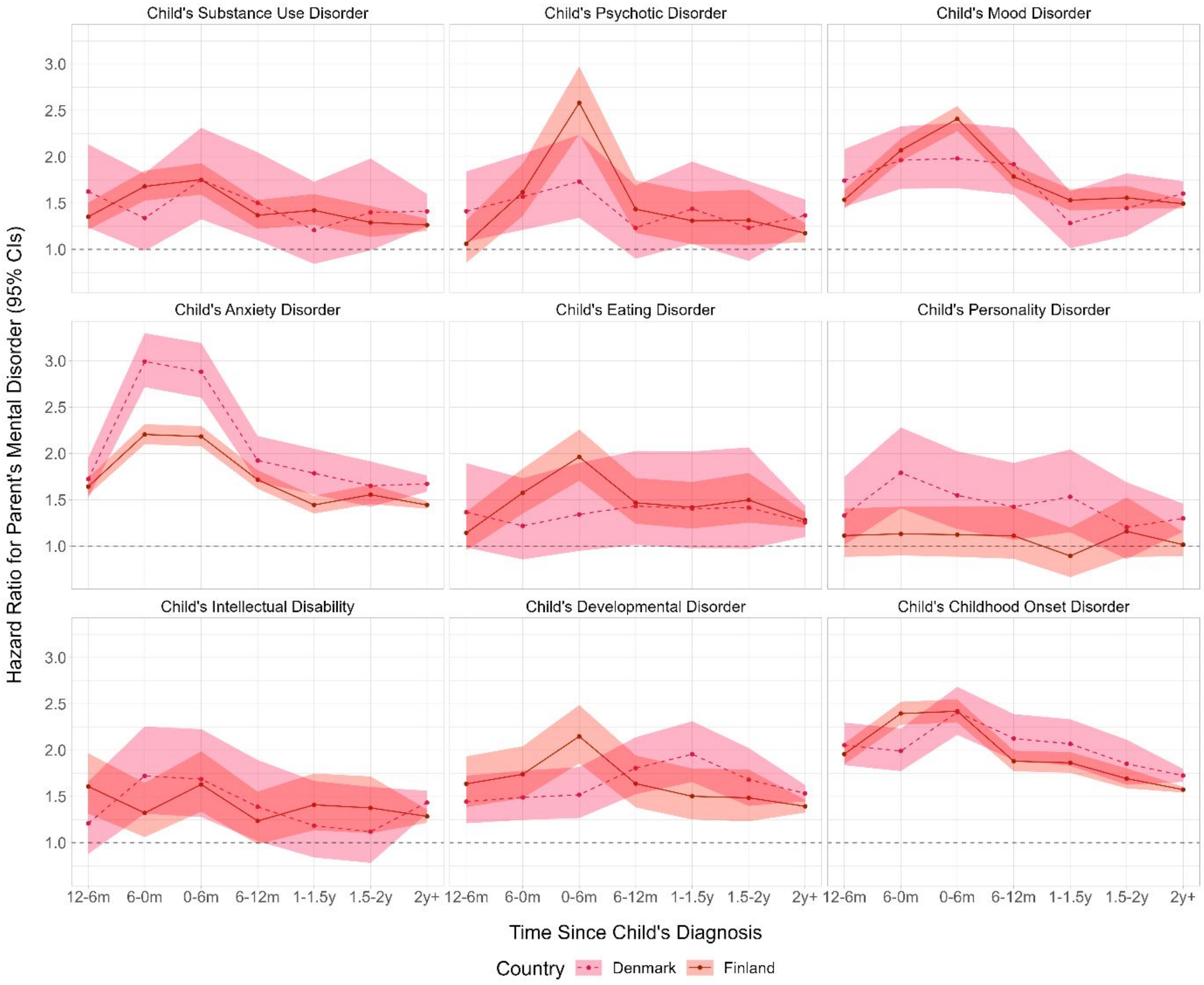

Supplement Figure 2. Women's Hazard Ratios and 95% Confidence Intervals of Mental Disorder in Relation to a Child's Specific Mental Disorder Before and After the Child's Diagnosis

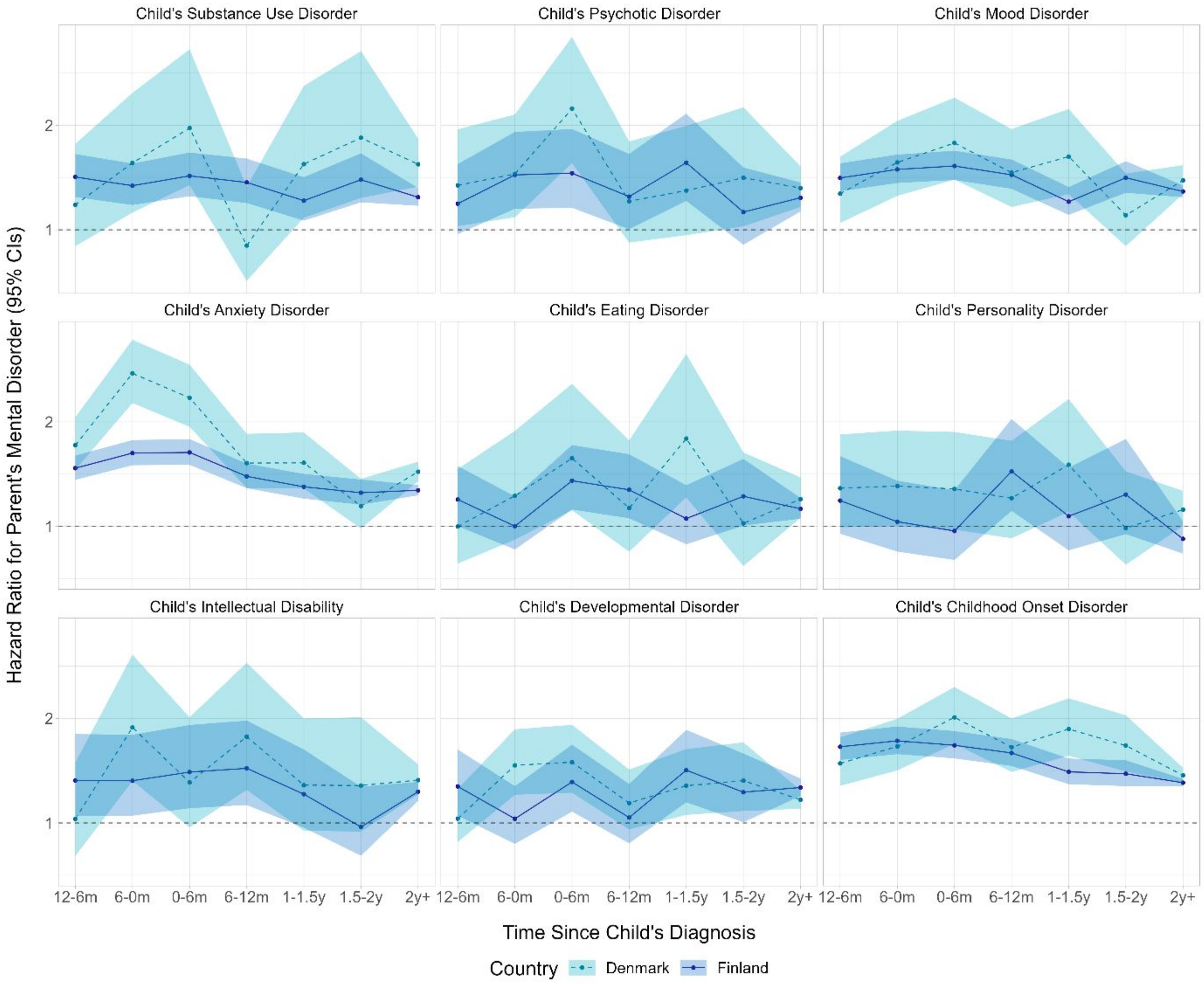

Supplement Figure 3. Men's Hazard Ratios and 95% Confidence Intervals of Mental Disorder in Relation to a Child's Specific Mental Disorder Before and After the Child's Diagnosis

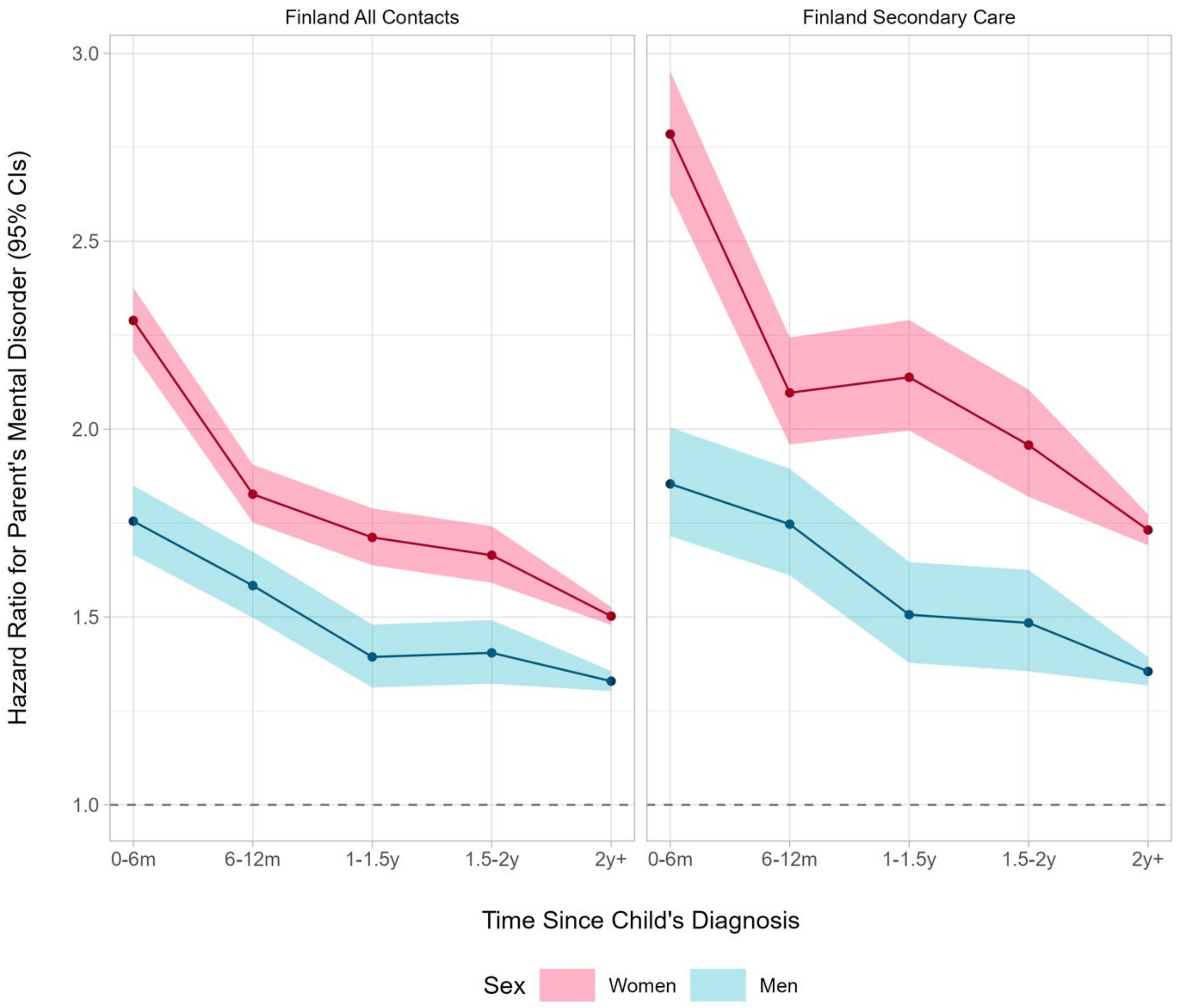

Supplement Figure 4. Hazard Ratios and 95% Confidence Intervals of Parents' Mental Disorder in Relation to Any Mental Disorder After the Child's Diagnosis in Finland

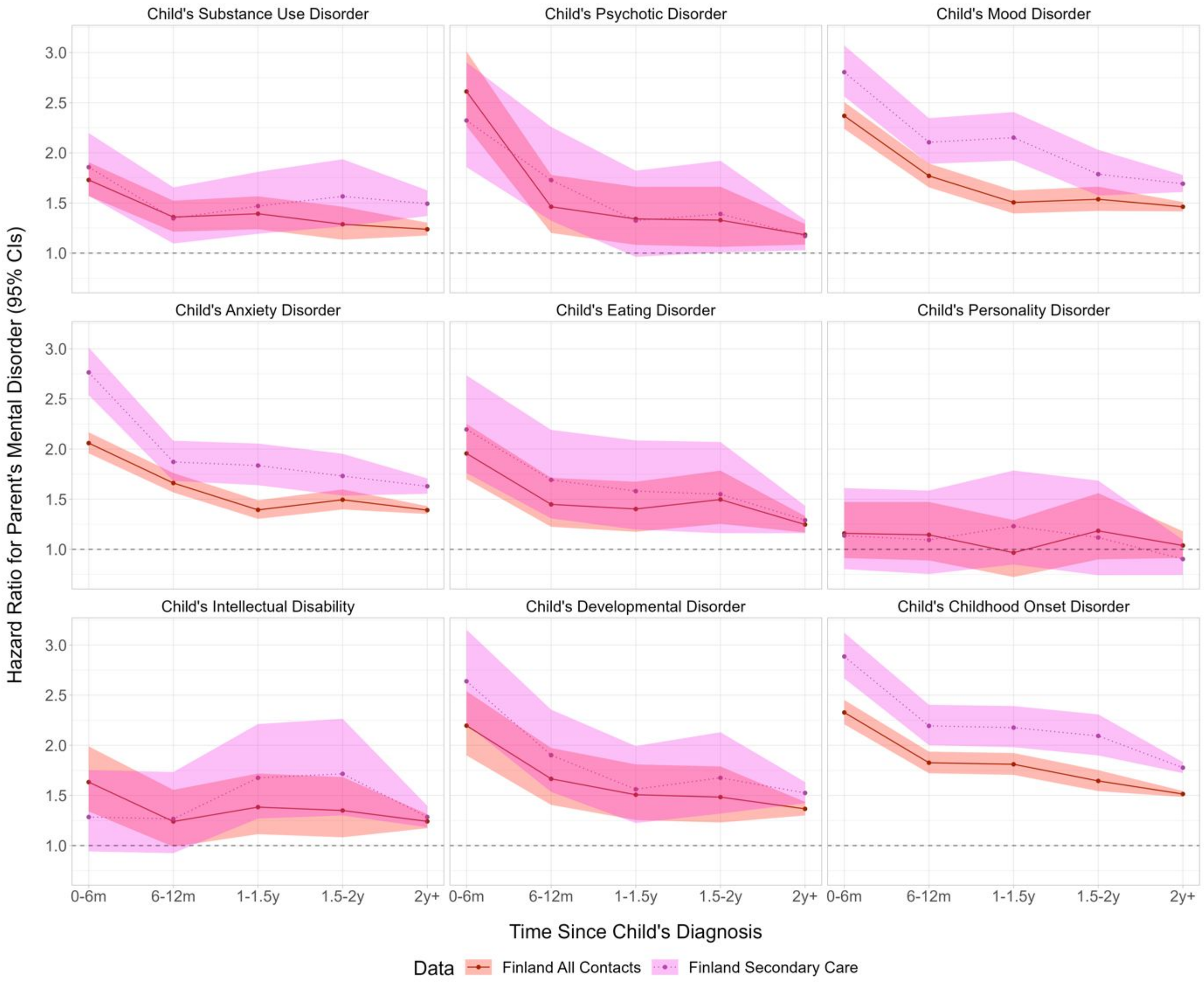

Supplement Figure 5. Women's Hazard Ratios and 95% Confidence Intervals of Mental Disorder in Relation to a Child's Specific Mental Disorder After the Child's Diagnosis in Finland

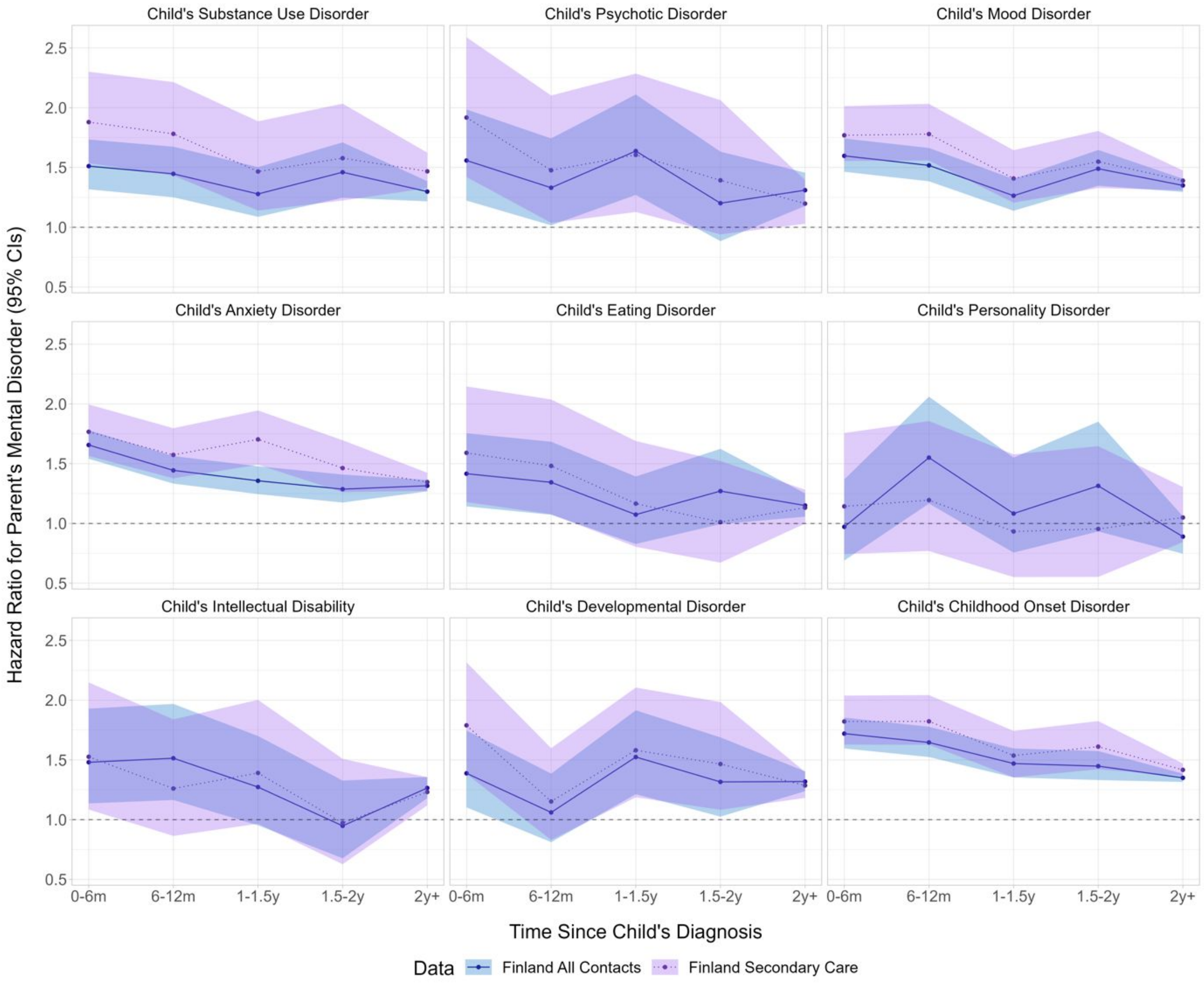

Supplement Figure 6. Men's Hazard Ratios and 95% Confidence Intervals of Mental Disorder in Relation to a Child's Specific Mental Disorder After the Child's Diagnosis in Finland
